# Machine learning-driven optimization of asphalt performance using eco-friendly waste modifiers

**DOI:** 10.1038/s41598-026-51700-x

**Published:** 2026-05-12

**Authors:** Priyam Nath Bhowmik, Kezia Saini, Pradyut Anand, Prachi Kushwaha, Pragya Prakash, Sanchit Anand

**Affiliations:** 1 Department of Civil Engineering, VNR Vignana Jyothi Institute of Engineering and Technology, Hyderabad, Telangana 500118 India; 2 Department of Mathematics, Madanapalle Institute of Technology and Science, Kurabalakota, Andhra Pradesh 517325 India; 3 Department of Civil Engineering, School of Engineering and Technology, Noida International University, Greater Noida, Uttar Pradesh 201310 India; 4 Department of Civil Engineering, Graphic Era (Deemed to be University), Dehradun, 248002 India; 5 Department of Civil Engineering, Nirma University, Ahmedabad, Gujarat 382481 India; 6 Government Engineering College, Banka, Bihar 813102 India; 7 Department of Civil Engineering, Manipal University Jaipur, Jaipur, Rajasthan 303007 India

**Keywords:** Complex shear modulus, Dynamic creep performance, Environmental sustainability, Indirect tensile strength (ITS), Life-cycle costs, Sustainable construction, Energy science and technology, Engineering, Environmental sciences, Materials science

## Abstract

Sustainable road construction is crucial in minimizing energy consumption, greenhouse gas emissions, and depletion of natural resources. Traditional asphalt production practices are under scrutiny due to their environmental impact, necessitating a shift towards sustainable technologies. Incorporating waste materials like Low-Density Polyethylene (LDPE) and Wood Apple Shell (WAS) powder in asphalt can improve mechanical properties, reduce material costs, and contribute to sustainability by reducing reliance on virgin materials and diverting waste from landfills. This research investigates the effects of these modifiers on asphalt properties, durability, and environmental impact, offering insights into eco-friendly practices for sustainable road infrastructure without conducting a full environmental life cycle assessment. The comprehensive analysis of LDPE + WAS modified asphalt mixtures identifies the 6% dosage as optimal, demonstrating superior performance across mechanical properties, moisture resistance, and economic viability. The mixture achieves enhanced tensile strength and stability, with a dry ITS of 2.1 MPa and a stability of 30.0 kN, indicating improved load-bearing capacity. It also shows excellent moisture resistance with a TSR of 90%, crucial for extending pavement life. Economically, the 6% dosage offers the lowest life-cycle costs and reduced maintenance expenses, making it a cost-effective choice. The use of waste materials contributes to sustainability, while improved dynamic creep performance and complex shear modulus enhance durability under heavy traffic. These findings underscore the innovative use of LDPE + WAS in asphalt mixtures, offering significant advancements in performance, cost-effectiveness, and sustainability for road construction projects.

## Introduction

Sustainable road construction has become a pressing concern in the global infrastructure landscape^1^. The need to reduce energy consumption, greenhouse gas emissions, and the depletion of natural resources has led researchers to explore innovative approaches to asphalt pavement design and production^2,3^. The global demand for robust and sustainable infrastructure has placed significant pressure on conventional road construction practices^4,5^. Asphalt pavements, a cornerstone of modern transportation networks, are facing scrutiny due to their environmental footprint and reliance on finite natural resources^6^. Traditional asphalt production consumes significant energy, generates greenhouse gas emissions^7,8^, and relies heavily on petroleum-based bitumen, a dwindling fossil fuel. This scenario necessitates a paradigm shift towards sustainable asphalt pavement technologies that minimize environmental impact while ensuring long-lasting performance^3,9,10^.

One promising avenue in this pursuit involves incorporating waste materials into asphalt mixtures^9^. This approach aligns with the principles of circular economy and resource management, transforming waste streams into valuable engineering materials^1–3,11^. Among the diverse array of potential waste materials, wood apple shell and low-density polyethylene have emerged as promising candidates for asphalt modification^12^.

Wood apple (*Limonia acidissima*) is a tropical fruit tree native to the Indian subcontinent and Southeast Asia. While the fruit’s pulp finds use in food and traditional medicine, the hard, woody shell is often discarded as agricultural waste^13,14^. This abundant biomass, rich in lignin and cellulose, exhibits potential as a sustainable filler or modifier in asphalt mixtures. Studies have shown that incorporating Wood apple shell (WAS) into asphalt can enhance its strength, stiffness, and resistance to moisture damage^15–18^.

Similarly, Low Density Polyethylene (LDPE), a ubiquitous thermoplastic commonly used in packaging, represents a significant environmental challenge due to its persistence in landfills^19–21^. However, its inherent durability, flexibility, and resistance to degradation make it an attractive candidate for asphalt modification. Incorporating LDPE into asphalt can improve its flexibility, fatigue resistance, and resistance to cracking, ultimately extending the pavement’s service life^22–25^. The utilization of Wood Apple Shell and Low-Density Polyethylene in asphalt modification presents a multifaceted approach to enhancing sustainability^26^. This approach conserves natural resources by reducing the demand for virgin aggregates and bitumen, thereby minimizing the environmental impact associated with their extraction and processing. Furthermore, it provides a sustainable waste management solution by diverting WAS and LDPE from landfills and mitigating their associated hazards^27^. The unique properties of WAS and LDPE have the potential to enhance the mechanical and rheological properties of asphalt mixtures, leading to improved durability, longevity, and reduced maintenance requirements^28–30^. Lastly, utilizing these waste materials as asphalt modifiers can potentially reduce material costs compared to conventional options, offering economic advantages for road construction projects^9,31^.

This research aims to comprehensively investigate the potential of utilizing WAS and LDPE as sustainable modifiers in asphalt mixtures. The study delves into the optimal processing techniques for WAS and LDPE, their combined effects on asphalt properties, and the development of mixed designs optimized for performance and sustainability. This investigation was carried out by a systematic experimental program to evaluate the influence of WAS and LDPE on key asphalt performance indicators. The evaluation includes mechanical properties such as Marshall stability, flow, bulk density, voids in mineral aggregate. Moreover, the research evaluates durability characteristics, including moisture susceptibility, Indirect Tensile Strength, Complex Shear Modulus, Dynamic Creep Performance and fatigue performance. Finally, a life cycle assessment was conducted to quantify the environmental benefits of utilizing WAS and LDPE in asphalt compared to conventional mixtures. The findings of this research contribute to the growing body of knowledge on sustainable asphalt pavement technologies, providing valuable insights for engineers, policymakers, and stakeholders seeking to minimize the environmental footprint of road infrastructure while ensuring its long-term performance and cost-effectiveness.

While several previous studies have applied machine learning techniques to asphalt mixture analysis, this work distinguishes itself through a holistic integration of eco-friendly modifiers and multi-objective optimization^32–36^. Specifically, the use of LDPE and WAS—both sustainable, waste-derived materials combined with machine learning-driven prediction and life cycle cost analysis provides a broader performance-economics perspective than conventional ML studies. Unlike prior research that primarily focused on predicting mechanical properties, the present framework synthesizes experimental data, cost modeling, and optimization into a single decision-making tool. This integrated approach not only enhances the predictive capacity but also supports practical implementation of sustainable asphalt design.

The manuscript is structured as follows: Section  “Materials and methods” presents the materials used and the experimental program, detailing sample preparation and testing protocols. Section “Results and discussion” discusses experimental results, including mechanical properties, durability, fatigue performance, and rheological behavior, followed by a life cycle cost analysis. Section “Machine learning and multi-objective optimization for performance and cost prediction” introduces the machine learning models and multi-objective optimization strategies applied to the dataset. Finally, section “Conclusion” presents the conclusions and outlines future research directions.

## Materials and methods

### Materials

**Fig. 1:**
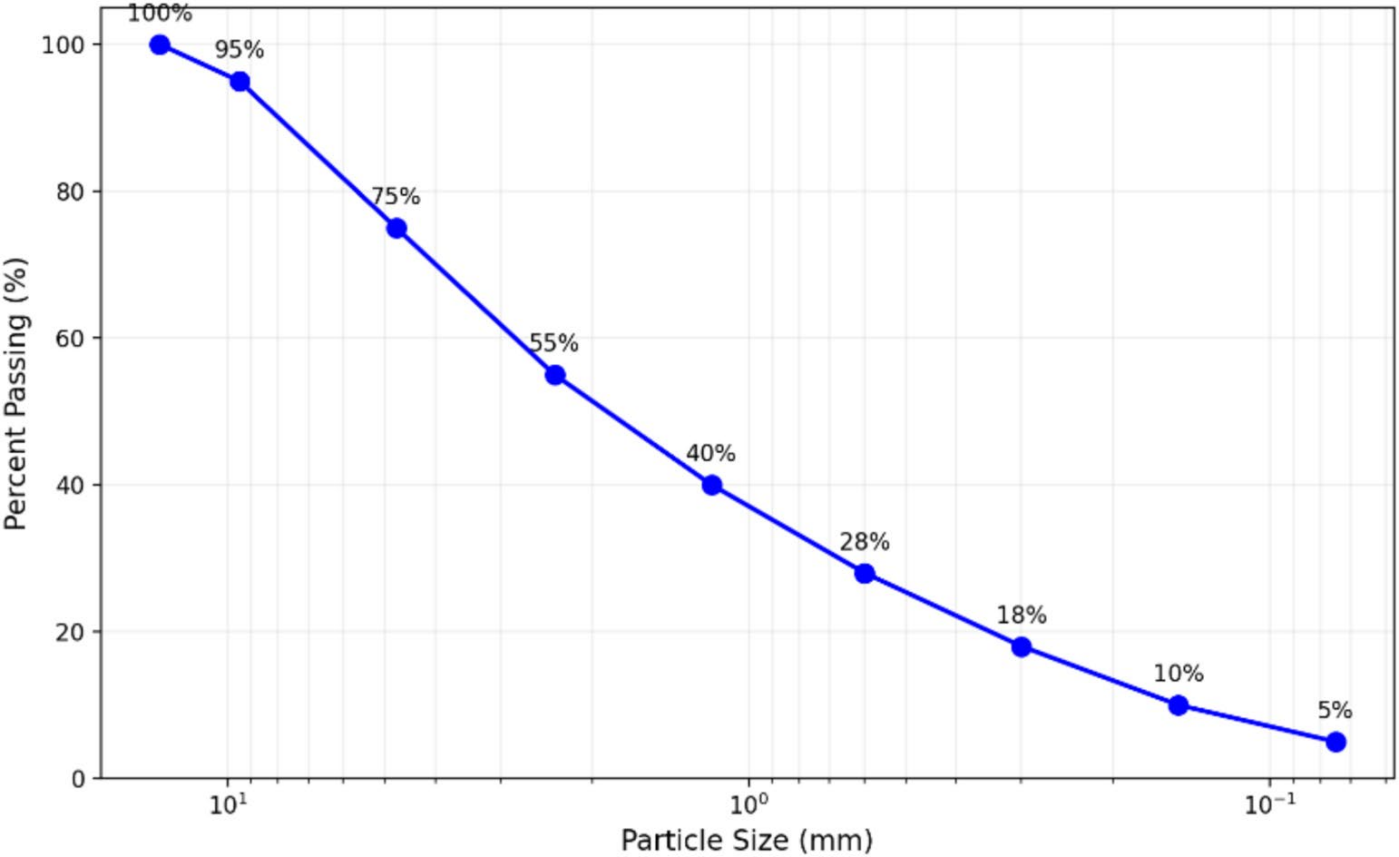
Gradation of aggregates as per IRC 37:2018.

Conventional VG 30 grade asphalt binder was used in this study. The bitumen was procured from Bitumix India LLP, a commercial supplier from Sunderpur, Guwahati, Assam, India. Aggregates used in this study were crushed stone brought from a nearby quarry owned by PLR Construction, Madanapalle, Andhra Pradesh, India. Nine different aggregate sizes, ranging from 13.2 mm to 0.075 mm (Fig. 1) were used in the study.

**Fig. 2:**
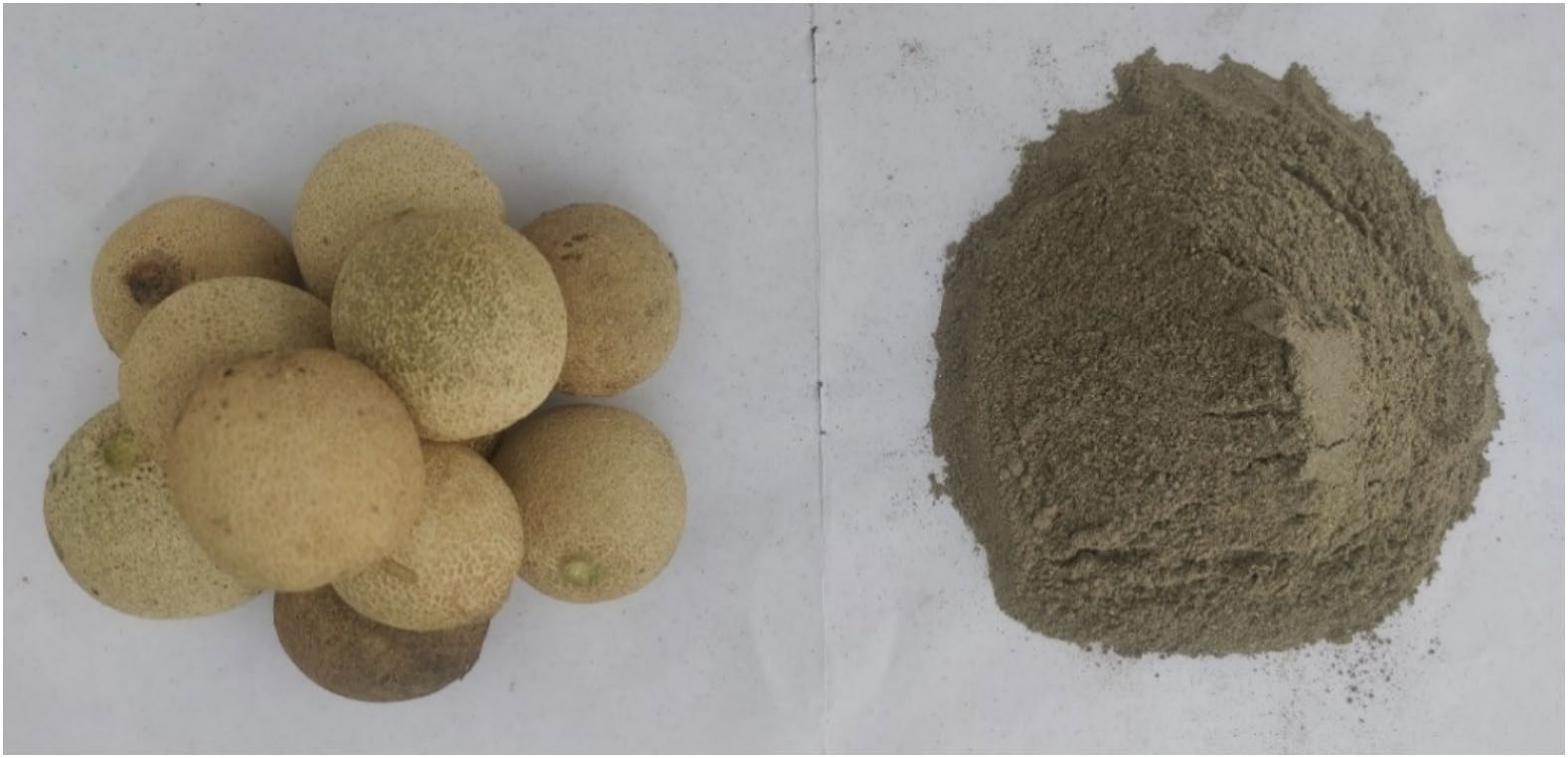
Wood Apple shell powder used in the study.

Wood apple is a tropical fruit tree native to the Indian subcontinent and Southeast Asia. The fruit itself has a pulp that is used in food and traditional medicine. The wood apple shell is the hard, outer covering of the fruit. This shell is often discarded as agricultural waste. However, it has shown potential as a sustainable material in various applications, including asphalt modification. This study uses the wood apple shell obtained from the local market of Batasingaram, Telangana India as shown in Fig. 2. The collected shells were cleaned, dried, and then crushed into a fine powder to be incorporated into the bituminous mix. The resulting WAS powder had an average particle size of approximately 150 μm, ensuring sufficient surface area for effective interaction with the binder. Based on the preliminary chemical screening, the powder was found to be rich in lignocellulosic components, particularly lignin (~ 32%) and cellulose (~ 38%), which contribute to improved stiffness and moisture resistance when incorporated into asphalt. The fibrous microstructure of WAS enhances its adhesion with the bitumen matrix, thereby reinforcing the mechanical performance and durability of the asphalt mix.

**Fig. 3.**
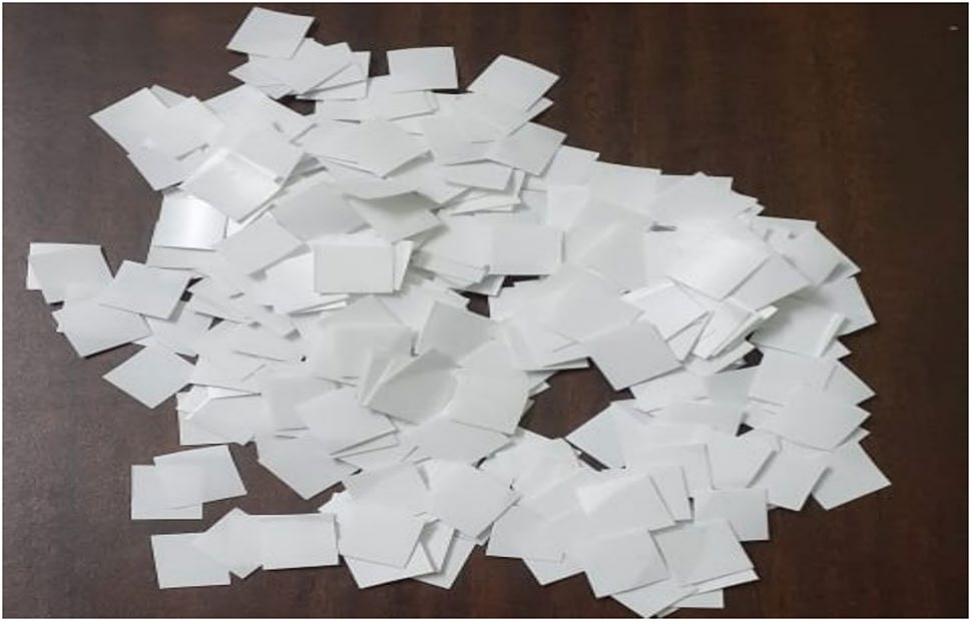
2 × 2 cm cut pieces of LDPE used in the study.

Polyethylene is a long-chain hydrocarbon derived from the polymerization of ethylene. It is a relatively inexpensive material that exhibits thermodynamic instability and a crystalline structure, characteristics of a plastomer. Low Density Polyethylene (LDPE) is a type of plastic commonly used in packaging, particularly for items like plastic bags, shrink wrap, and some food containers. In this study plastic carry bags (cut into 2 × 2 cm pieces) as shown in Fig. 3 have been used. The choice of 2 × 2 cm LDPE particle size was based on both literature recommendations and preliminary lab trials. This size facilitated good melting and uniform dispersion when mixed at elevated temperatures. Smaller particle sizes, although considered, led to operational difficulties such as sticking and clumping during blending. On the other hand, larger sizes impeded homogeneous mixing and resulted in inconsistencies in binder modification. The 2 × 2 cm size offered an effective balance between ease of handling and efficient integration into the binder matrix, contributing to consistent performance enhancements across mechanical and durability parameters. While this size was optimal under the present laboratory conditions, future studies could explore a broader size spectrum to further refine performance outcomes.

### Experimental program

**Fig. 4:**
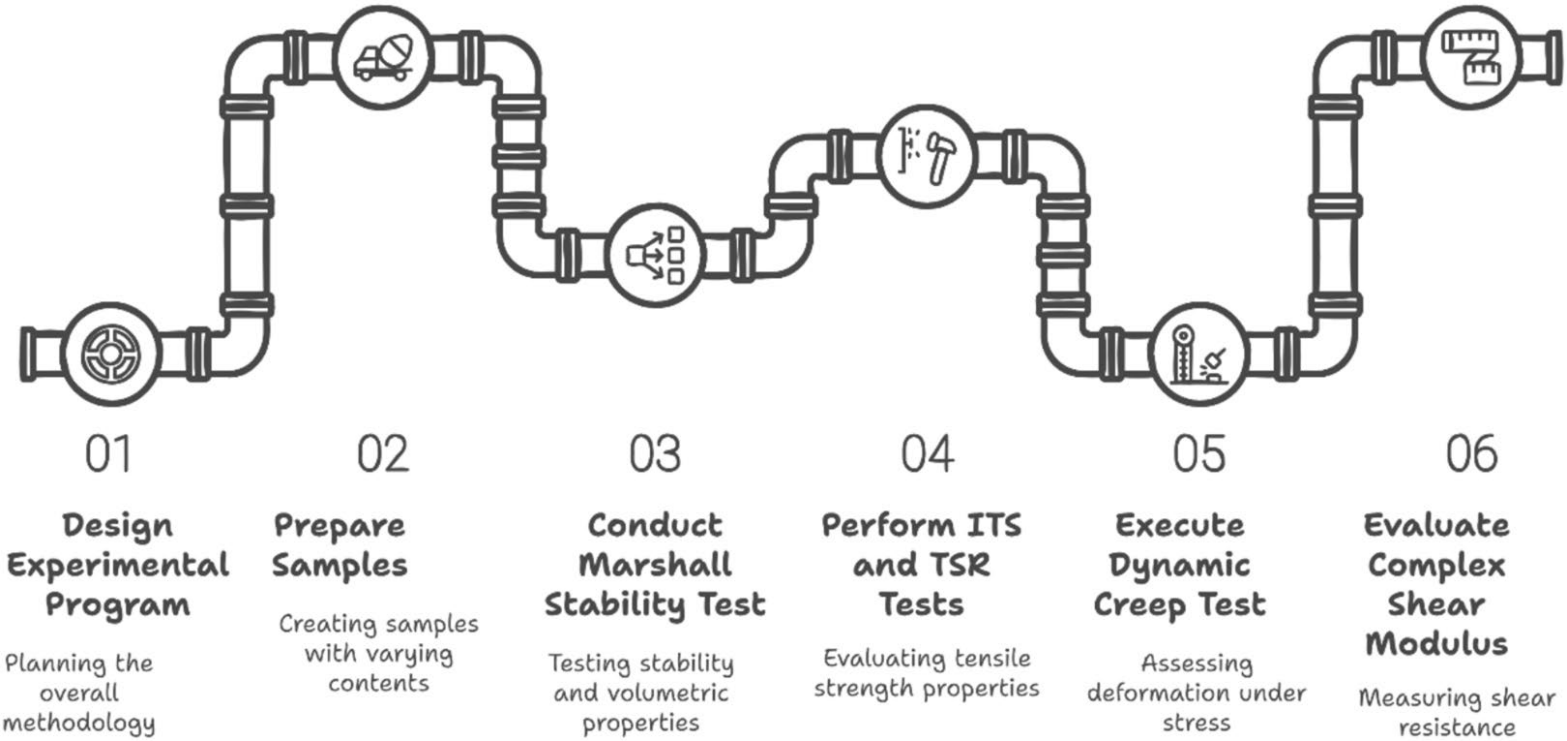
Experimental program visualization.

The experimental program was designed to evaluate the mechanical and durability performance of asphalt mixtures modified with LDPE and WAS and is shown in Fig. 4. The overall methodology followed is illustrated in Figure X (experimental workflow). The program included the preparation of samples at varying LDPE + WAS contents (2%, 4%, 6%, 8%, and 10%) and testing the following properties:

- *Marshall stability and volumetric analysis* Conducted as per ASTM D6927. Three specimens were tested for each mix composition.
- *Indirect tensile strength (ITS) and tensile strength ratio (TSR)* Performed according to AASHTO T283. A total of six specimens per content level were tested (three dry, three wet-conditioned).
- *Dynamic creep test* Conducted at 40 °C using a sustained stress level of 100 kPa in accordance with AASHTO T378. Three replicates per mix type were tested.
- Complex shear modulus (G*) Evaluated using a Dynamic Shear Rheometer (DSR) at 60 °C across frequencies ranging from 0.1 to 10 Hz as per AASHTO T315.
- *Fatigue life estimation* Performed using a four-point bending beam test protocol. Prediction models were calibrated based on performance at five strain levels using three specimens per content.
- *Life cycle cost analysis* Estimated using initial cost breakdowns and maintenance projections based on industry schedules and literature-based assumptions.

The tests were conducted in a controlled lab environment at the Civil Engineering Laboratory of the campus. The sample preparation process involved the controlled incorporation of LDPE and WAS modifiers into the asphalt binder. The VG 30 bitumen was preheated to 160 °C, while the LDPE (2 × 2 cm pieces) and WAS powder were added at a 1:1 ratio by weight, based on the total modifier content. LDPE was gradually introduced into the binder and stirred continuously for 20 min at 160–165 °C to ensure adequate melting and dispersion. Subsequently, the WAS powder was added and blended uniformly for another 10 min. The mixing process was performed using a high-shear mechanical stirrer at 1000 rpm, ensuring homogenous dispersion of both modifiers within the binder matrix. Homogeneity was verified through visual inspection for particle clustering and binder streaks, and spot-sampling to confirm uniform distribution. Modified binders were then mixed with preheated aggregates and compacted using a Marshall compactor or gyratory compactor, depending on the test requirement. This preparation protocol was applied consistently across all dosages to maintain reproducibility and accuracy of test results.

## Results and discussion

### Experimental results

The experimental results comprehensively analyzed various parameters to determine the optimal binder and modifier content for enhanced bituminous mixtures. The determination of Optimum Binder Content (OBC) revealed that 5.5% bitumen content provides maximum stability, ensuring a balance between strength and flexibility. The study further examined the incorporation of LDPE and WAS powder, with 6–8% identified as the ideal range for improving stability, reducing void ratios, and enhancing volumetric properties. Indirect Tensile Strength (ITS) peaked at 8% modifier content, demonstrating improved particle binding and aggregate-binder interactions, while moisture susceptibility tests showed that the 6–8% range achieved optimal Tensile Strength Ratio (TSR) values, indicating excellent resistance to moisture damage. Strength evolution analysis reinforced this finding, with minimal strength loss observed at 6–8% content. Additionally, fatigue performance evaluations highlighted 6% as the most effective content, offering the highest resistance to failure under cyclic loading. Dynamic creep analysis confirmed that 6% content minimized permanent deformation, while viscoelastic behavior studies demonstrated balanced stiffness and flexibility at this content. Finally, a life cycle cost analysis identified 6% LDPE + WAS content as the most cost-effective option, achieving the lowest overall cost and the longest lifespan. Collectively, these results underscore the effectiveness of the 6–8% range, particularly 6%, as the optimal modifier content for sustainable and durable bituminous mixtures.

#### Determination of optimum binder content (OBC)

**Fig. 5:**
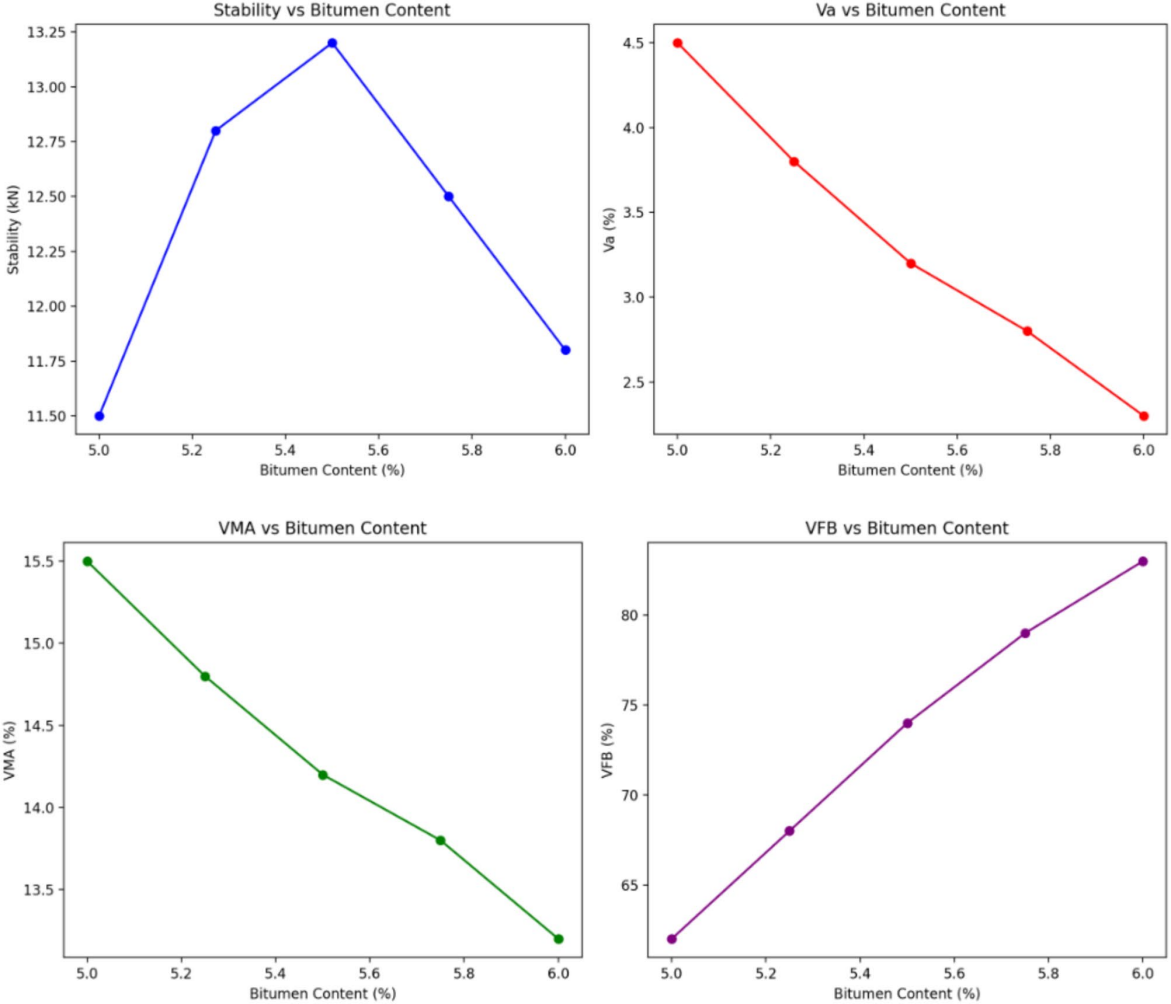
Variation of stability and volumetric parameters for the conventional bituminous mix.

The Marshall mix design method was utilized to determine the optimal binder content for the conventional bituminous mixture. The graph presented in Fig. 5 depicts the variation in Marshall Stability Value with different bitumen content percentages, ranging from 5.25 to 6%, (5.25, 5.5, 5.75 and 6%), at 0.25% intervals, in an asphalt mix. The stability value, represented by the blue bars and emphasized by the red trend line, peaks at 5.5% bitumen content, reaching a maximum value of 13.2 kN, indicating optimal load-bearing capacity. At lower and higher bitumen contents, the stability values decrease, reaching 11.5 kN, 12.8 kN, 12.5 KN and 11.8 kN, respectively (Table 1). This trend suggests that the asphalt mix exhibits the greatest stability at 5.5% bitumen content, striking a balance between strength and flexibility, whereas insufficient or excessive bitumen reduces stability, likely due to inadequate binding or excessive flexibility. While the Volumetric Fill with Bitumen (VFB) continues to increase beyond 5.5%, selecting this value as the baseline ensures the highest load-bearing capacity as indicated by the peak in Marshall Stability. Opting for a higher bitumen content would lead to excessive binder, which can reduce stiffness, increase susceptibility to rutting, and compromise the mixture’s structural integrity. Therefore, 5.5% was chosen to maintain an optimal balance between strength, durability, and volumetric performance. The analysis was essential for determining the optimal bitumen content to maximize the durability and performance of asphalt pavements.

**Table 1:** Stability values and volumetric parameters of the un-modified bituminous mix.

Bitumen content (%)	Stability (kN)	Va (%)	VMA (%)	VFB (%)
5	11.5	4.5	15.5	62
5.25	12.8	3.8	14.8	68
5.5	13.2	3.2	14.2	74
5.75	12.5	2.8	13.8	79
6	11.8	2.3	13.2	83

#### Ideal blend of LDPE and WAS powder for enhanced bituminous mixture

**Fig. 6:**
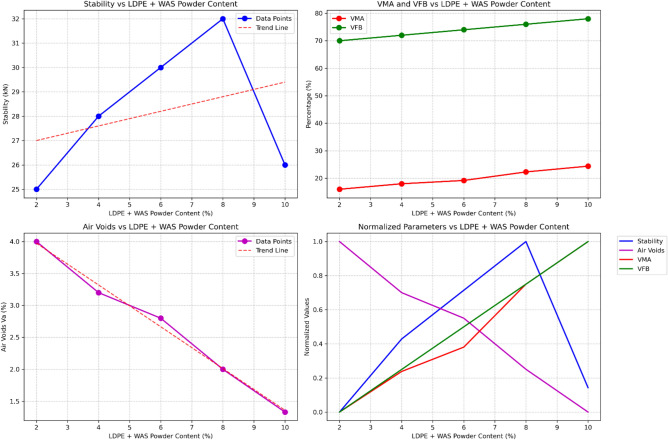
Comprehensive analysis of asphalt mix parameters with LDPE + WAS powder content.

The study investigated the optimal percentages of LDPE and WAS powder that yielded the maximum stability in bituminous mixtures. The bituminous mix was prepared by blending VG 30 grade bitumen with LDPE (using 2 × 2 cm cut pieces) and WAS powder in a 1:1 ratio. The 1:1 LDPE:WAS ratio was established through a preliminary screening phase in which three compositional ratios 2:1 (LDPE-dominant), 1:1 (equal) and 1:2 (WAS-dominant) were evaluated at a fixed total modifier content of 6% using Marshall Stability as the primary selection criterion. The 2:1 ratio produced mixtures with higher flow values and reduced stability (approximately 24.8 kN), attributable to excess thermoplastic softening of the binder matrix. The 1:2 ratio yielded improved stability but exhibited workability difficulties during blending, including visible WAS agglomeration and non-uniform dispersion as verified by spot-sampling. The 1:1 blend consistently achieved the highest stability (30.0 kN), uniform modifier distribution and acceptable workability across the preparation protocol. It also ensured functional complementarity: LDPE contributes thermoplastic flexibility and fatigue resistance, while WAS provides lignocellulosic stiffening and moisture resistance creating a synergistic performance enhancement. It is acknowledged that a full factorial optimization of the LDPE: WAS compositional ratio conducted independently across multiple dosage levels represents a logical extension of the present work and is recommended as a priority for future investigation. The present study intentionally constrained the compositional variable to focus experimental resources on the dosage-performance optimization framework, which constitutes the primary scientific contribution. The LDPE and WAS powder mix was incorporated into the bituminous mix at five different percentages: 2%, 4%, 6%, 8%, and 10% by weight of the bitumen. The comprehensive analysis of the asphalt mix design incorporating LDPE + WAS powder reveals several significant trends and correlations across multiple parameters. As the LDPE + WAS powder content increases from 2 to 10%, the stability of the mix shows a strong positive linear correlation, increasing from 25 to 32 kN, indicating enhanced structural strength and load-bearing capacity. The maximum stability was achieved at a dosage of 8% and showed a falling trend thereafter. Simultaneously, the void-related parameters demonstrate notable changes: the Voids in Mineral Aggregates (VMA) exhibit a substantial increase from 16 to 24.4%, while the Voids Filled with Bitumen (VFB) show a steady rise from 70 to 78%, suggesting improved bitumen distribution within the mix (Table 2). Notably, there’s a significant inverse relationship with Air Voids, which decrease markedly from 4% to 1.33%, indicating that the LDPE + WAS powder effectively fills the void spaces in the mixture. The normalized parameter comparison further emphasizes these relationships, clearly illustrating (Fig. 6) how stability, VMA, and VFB increase proportionally while air voids decrease with increasing powder content. This pattern suggests that the incorporation of LDPE + WAS powder leads to a more densely packed, stable mixture with enhanced void-filling properties, while maintaining optimal bitumen content at 5.5%. These results collectively demonstrate the effectiveness of LDPE + WAS powder as a modifier in improving the structural and volumetric properties of the asphalt mixture.

**Table 2:** Variation of the stability for the combined bituminous mix.

Dosage of LDPE + WAS powder (%)	Stability (kN)	Va (%)	VMA (%)	VFB (%)
2	25	4	16	70
4	28	3.2	18	72
6	30	2.8	19.2	74
8	32	2	22.3	76
10	26	1.33	24.4	78

Furthermore, although 8% dosage demonstrated peak values in specific indicators like stability and ITS, a holistic evaluation across all performance domains, including fatigue life, dynamic creep resistance, moisture susceptibility (TSR), and economic efficiency confirmed that 6% LDPE + WAS content offers the most balanced and optimal outcome. The 6% blend not only exhibited robust mechanical strength and durability but also minimized permanent deformation and maintenance costs over the life cycle. These findings were validated through multi-objective optimization (GA and MOPSO), reinforcing that the 6% dosage provides the best trade-off among competing performance and economic criteria, making it the most suitable choice for sustainable pavement design. In comparison with conventional polymer-modified asphalt binders, such as those incorporating Styrene–Butadiene–Styrene (SBS), the 6% LDPE + WAS modified mix shows promising results. While SBS binders are widely recognized for their excellent elasticity and resistance to temperature-induced cracking, the LDPE + WAS blend delivers comparable performance in terms of Marshall Stability, Indirect Tensile Strength, and moisture resistance. Moreover, the use of waste-derived modifiers enhances the sustainability profile of the mix, offering a cost-effective and environmentally conscious alternative that aligns with circular economy principles.

To provide a transparent, quantitative basis for the designation of 6% LDPE + WAS content as the globally optimal dosage, a normalized weighted composite performance index (CPI) was constructed. Each performance criterion was normalized to a 0–1 scale (where 1.0 represents the best observed value within the experimental range) using the formula: Normalized Score = (Value − Min)/(Max − Min) for benefit criteria (higher is better: Stability, Dry ITS, TSR, Fatigue Life, Effective Lifespan) and Normalized Score = (Max − Value)/(Max − Min) for cost criteria (lower is better: Dynamic Creep Strain, Total Life Cycle Cost). Weights were assigned based on relative design importance within the Indian pavement context: Stability (0.15), Dry ITS (0.15), TSR (0.20), Fatigue Life (0.15), Dynamic Creep inversed (0.15) and Total Life Cycle Cost inversed (0.20). The resulting Composite Performance Index (CPI) for each dosage level is presented in Table 10.

#### Evaluation of indirect tensile strength

**Fig. 7:**
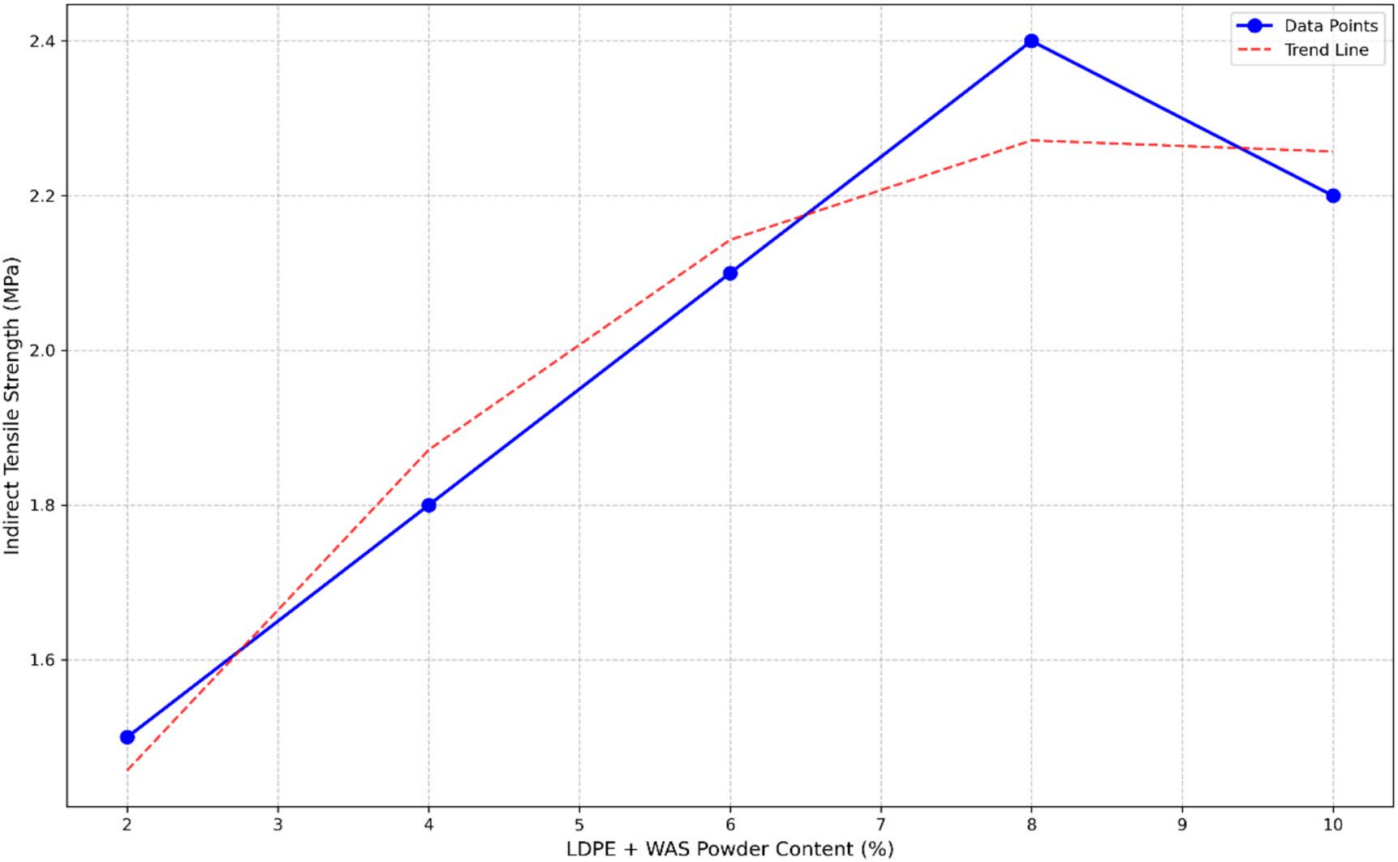
Effect of LDPE + WAS powder content on indirect tensile strength of modified asphalt mixture.

The indirect tensile strength (ITS) test is a fundamental mechanical characterization method widely used in asphalt mixture design to evaluate the material’s resistance to cracking and tensile deformation under loading conditions. This parameter is particularly crucial as it simulates the critical tensile stresses that develop at the bottom of the asphalt layer during service life under traffic loading and environmental conditions. In this study, the incorporation of Low-Density Polyethylene (LDPE) combined with Wood Apple Shell (WAS) powder as a modifier in asphalt mixtures was investigated at varying contents (2%, 4%, 6%, 8%, and 10% by weight) to assess their influence on the indirect tensile strength (Table 3). The initial increase in ITS values from 1.5 MPa at 2% content to 2.4 MPa content can be attributed to several mechanisms like enhanced particle binding due to the polymer’s adhesive properties, improved aggregate-binder interface strength, reduced void content leading to better compaction and increased cohesive strength of the modified binder. However, the subsequent decline in ITS to 2.2 MPa at 10% content suggests a threshold effect, where excessive modifier content begins to compromise the mixture’s performance (Fig. 7). This decrease could be attributed to factors such as over-saturation of the modifier, reduced workability affecting compaction efficiency, or potential agglomeration of particles leading to non-uniform distribution within the mixture. The observed peak at 8% content represents the optimal balance between these competing mechanisms, providing valuable guidance for mix design specifications in practical applications. This behavior aligns with fundamental principles of asphalt modification, where achieving an optimal modifier content is crucial for maximizing performance benefits while avoiding potential adverse effects from over-modification.

**Table 3:** Variation of indirect tensile strength with LDPE + WAS powder content.

Composite sample	LDPE + WAS powder content (%)	Indirect tensile strength (MPa)
1	2	1.5
2	4	1.8
3	6	2.1
4	8	2.4
5	10	2.2

The progressive increase in ITS up to the 8% threshold can be mechanistically explained by two concurrent phenomena. First, at processing temperatures of 160–165 °C, LDPE melts and forms a continuous thermoplastic film around aggregate surfaces, substantially improving the polymer–aggregate adhesion energy and reducing interfacial stress concentrations under tensile loading. Second, the lignocellulosic composition of WAS comprising approximately 38% cellulose and 32% lignin which introduces a network of micro-fibers that bridge incipient micro-cracks within the binder matrix acting analogously to short-fiber reinforcement in composite materials. The phenolic hydroxyl groups present in lignin also interact chemically with the polar components of VG 30 bitumen, forming hydrogen-bonded complexes that enhance cohesive strength. Beyond the 8% threshold, excess LDPE creates a discontinuous polymer phase that disrupts aggregate interlock, while agglomeration of WAS particles reduces the effective bonding surface area and introduces stress-concentrating discontinuities, collectively reducing tensile resistance.

**Fig. 8:**
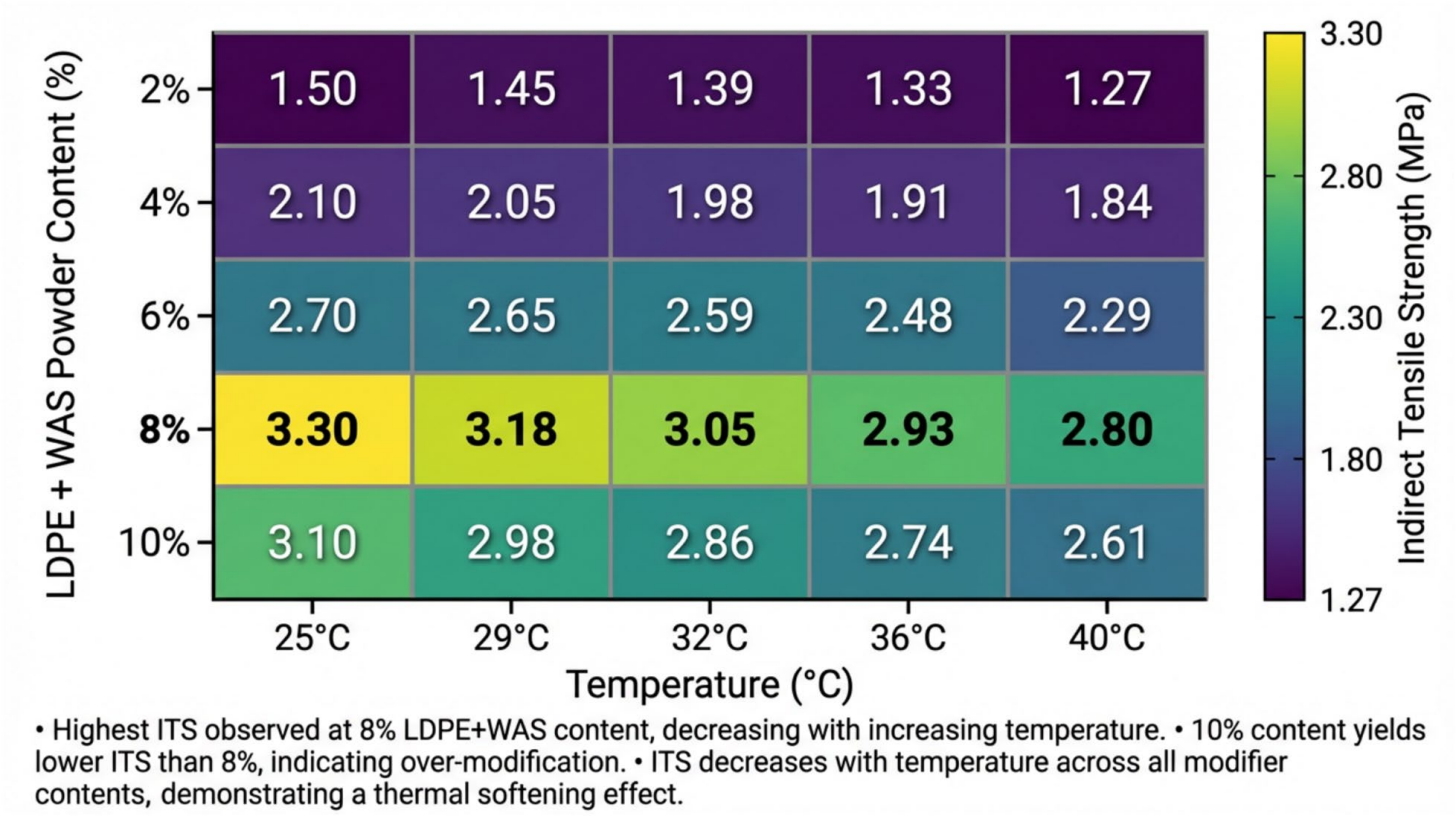
Heatmap of ITS with temperature and LDPE + WAS powder.

The Indirect Tensile Strength heatmap reveals a complex interplay between temperature, LDPE + WAS powder content, and resulting strength (Fig. 8). The map shows ITS values across temperatures (25–40 °C) and LDPE + WAS contents (2–10%). Higher ITS (yellow) is observed at lower temperatures (25 °C) across all LDPE contents, with strength consistently decreasing as temperature rises. Specifically, there’s a roughly 15% strength reduction from 25 °C to 40 °C. ITS increases with LDPE + WAS content up to 8%, peaking at 2.40 MPa (8% content, 25 °C), then declines to 10%. The lowest ITS (1.28 MPa) occurs at 2% content and 40 °C. Optimal performance (yellow zone) is found at 6–8% LDPE content and 25–30 °C, while moderate performance (green) spans 4–10% LDPE at moderate temperatures, and lower performance (blue) is seen at 2–4% LDPE and higher temperatures. This suggests an optimal mix design around 6–8% LDPE + WAS content, emphasizing temperature control during construction and service to maintain desired strength, as the mix exhibits temperature sensitivity. Over-dosing LDPE + WAS beyond 8% offers no added benefit.

This temperature-sensitive behavior has important implications for pavement performance in tropical climates, where surface temperatures frequently exceed 40 °C. The decline in ITS at elevated temperatures suggests a potential reduction in resistance to tensile cracking under thermal and traffic loads. Therefore, for regions with prolonged high-temperature exposure, additional measures, such as the use of temperature-resistant binder formulations, surface cooling techniques, or increased modifier content within optimal thresholds, may be necessary to ensure long-term structural integrity and functional performance of the pavement.

#### Evaluation of moisture susceptibility

**Fig. 9:**
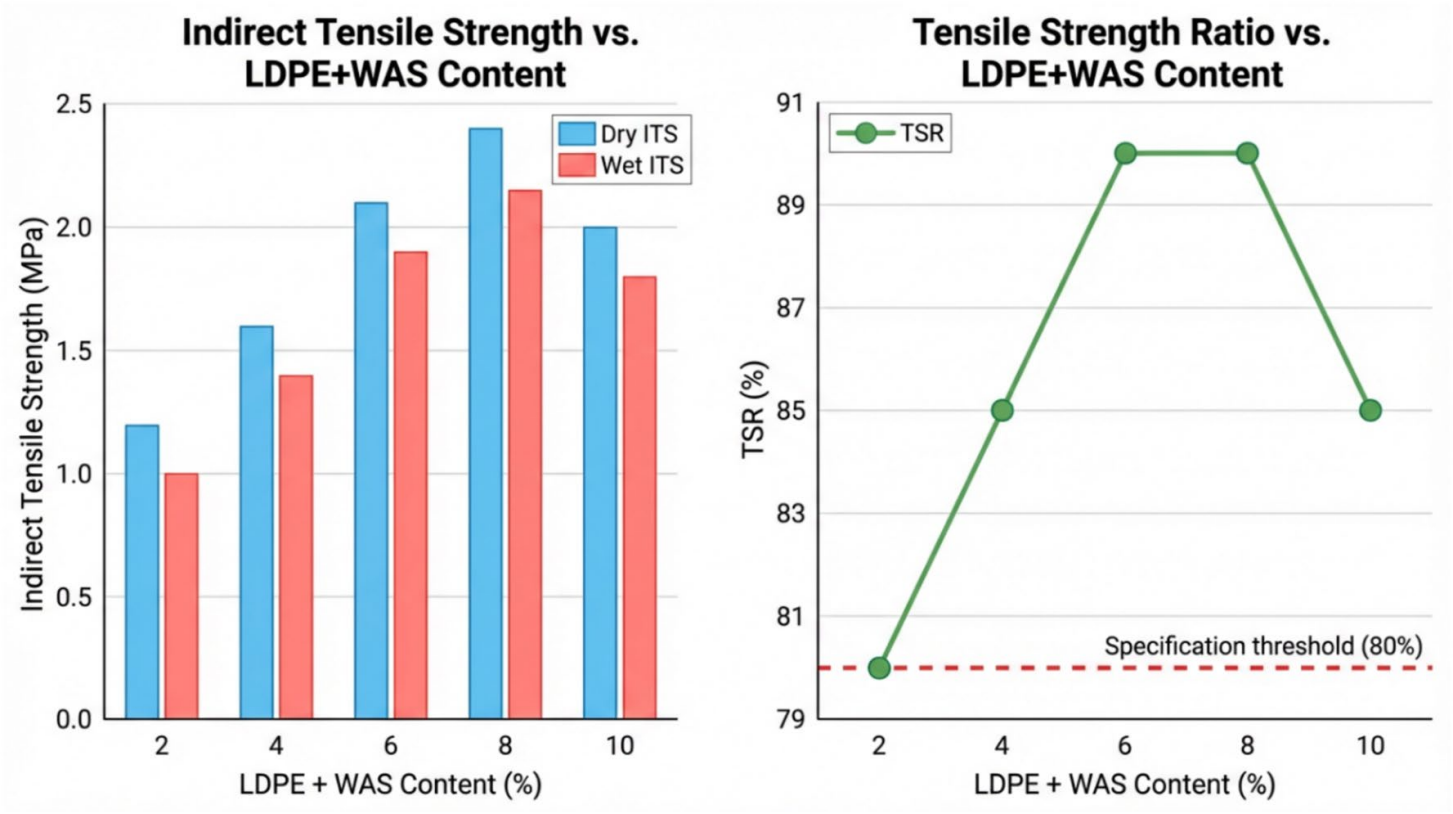
Tensile strength ratio and ITS analysis for LDPE + WAS modified Asphalt.

The Tensile Strength Ratio (TSR) is a crucial parameter in asphalt mixture design that evaluates the moisture susceptibility and durability of pavements under adverse environmental conditions. This test is performed according to AASHTO T283 specifications, where cylindrical specimens are prepared and divided into two subsets: one maintained in dry conditions and the other subjected to moisture conditioning through vacuum saturation (70–80% saturation level) followed by a freeze–thaw cycle as per AASHTO T283, where specimens were frozen at –18 °C for 16 h and then thawed in a 60 °C water bath for 24 h to simulate moisture-induced damage. The specimens undergo indirect tensile strength testing at 25 °C, where a compressive load is applied along the diametrical axis at a rate of 50 mm/minute until failure. The TSR is calculated as the ratio of the average tensile strength of moisture-conditioned (wet) specimens to that of unconditioned (dry) specimens, expressed as a percentage: TSR = (Wet ITS/Dry ITS) × 100. A minimum TSR value of 80% is typically specified by most highway agencies to ensure adequate resistance to moisture damage. Higher TSR values indicate better resistance to moisture-induced damage and improved long-term durability of the pavement. The analysis of the Tensile Strength Ratio (TSR) for asphalt mixtures with varying LDPE + WAS powder content reveals key insights into the material’s moisture susceptibility and performance. Table 4 and Fig. 9 illustrate that both Dry and Wet Indirect Tensile Strength (ITS) values peak at 8% LDPE + WAS content, with Dry ITS reaching 2.40 MPa and Wet ITS 2.16 MPa, indicating optimal structural integrity at this dosage. The TSR, which measures the ratio of Wet to Dry ITS, consistently exceeds the minimum required threshold of 80% across all content levels, demonstrating the mix’s resilience to moisture damage. TSR values improve with increasing LDPE + WAS content, peaking at 90% for 6–8% content, before slightly declining to 85% at 10% content. This suggests that while higher LDPE content enhances moisture resistance, excessive amounts may lead to diminishing returns. The analysis underscores the importance of optimizing LDPE + WAS content to balance strength and moisture resistance, with 6–8% identified as the optimal range for achieving maximum performance and durability in asphalt mixtures. The superior moisture resistance of the 6–8% LDPE + WAS modified mixtures can be attributed to complementary hydrophobic and chemical adhesion mechanisms. The continuous LDPE film, once formed at elevated mixing temperatures, imparts a hydrophobic coating on aggregate surfaces that physically impedes water ingress into the binder–aggregate interface, thereby reducing stripping susceptibility. Concurrently, the polar functional groups particularly the hydroxyl and ether linkages in WAS cellulose form strong electrostatic attractions with cationic surface sites on the crushed stone aggregates used in this study, providing chemically reinforced adhesion that persists under moisture conditioning. This dual-mechanism protection explains the consistent TSR values above 85% across all dosages and the peak of 90% at 6–8% content. The marginal decline at 10% content likely reflects disruption of the polymer film continuity due to phase separation at high modifier loadings, which creates preferential moisture diffusion pathways at the binder–aggregate interface.

**Table 4:** Comprehensive TSR and strength analysis.

Composite sample	LDPE + WAS content (%)	Dry ITS (MPa)	Wet ITS (MPa)	TSR (%)
1	2	1.5	1.2	80
2	4	1.8	1.53	85
3	6	2.1	1.89	90
4	8	2.4	2.16	90
5	10	2.2	1.87	85

#### Strength evolution analysis

Strength Evolution analysis is a comprehensive evaluation process that assesses the performance of asphalt mixtures with varying LDPE + WAS contents through systematic testing and analysis. The procedure begins with sample preparation, where specimens containing different percentages (2, 4, 6, 8, and 10%) of LDPE + WAS are compacted using a gyratory compactor, followed by testing under both dry and moisture-conditioned states according to AASHTO T283 specifications. The Indirect Tensile Strength (ITS) test is conducted at 25 °C with a loading rate of 50 mm/minute, where the maximum load is recorded and ITS is calculated using the formula ​ ITS = (2 P)/(π dt), where P is the maximum load (N), d is specimen diameter (mm), and t is specimen thickness (mm). The Tensile Strength Ratio (TSR) is then determined as $TSR=\frac{Wet ITS}{Dry ITS}\times 100$.

The collected data (Table 5) is visualized through plots showing dry ITS, wet ITS, and normalized TSR trends, with a gray-shaded area representing the strength loss zone. The analysis evaluates strength development, moisture susceptibility, and TSR evolution, considering criteria such as higher ITS values for better structural capacity, smaller gaps between dry and wet ITS for improved moisture resistance, and TSR values exceeding 80% for acceptable moisture resistance. Quality control measures include multiple specimen testing, statistical analysis, and result validation against standard specifications, ultimately providing crucial insights for optimizing mix design, predicting field performance, ensuring durability requirements, understanding performance limitations, and establishing quality control parameters for determining the optimal LDPE + WAS content that balances both strength and moisture resistance requirements in the modified asphalt mixture. The diminishing moisture-induced strength loss (narrowing gap between Dry and Wet ITS) as dosage increases from 2 to 6% reflects the progressive consolidation of the polymer–lignocellulosic network, additional modifier content increases the density of hydrophobic LDPE bridges and polar WAS anchor points collectively reducing the effective moisture diffusion area at critical interfaces. The reversal above 8% is consistent with a percolation-threshold effect, wherein excessive filler loading increases tortuosity and internal void discontinuities, paradoxically providing new sites for moisture retention.

**Table 5:** Comprehensive strength and performance analysis of LDPE + WAS modified asphalt mixtures.

LDPE + WAS content (%)	Dry ITS (MPa)	Wet ITS (MPa)	TSR (%)	Strength loss (%)	Moisture resistance (%)	Performance Index
2	1.5	1.2	80	20	80	1.08
4	1.8	1.53	85	15	85	1.415
6	2.1	1.89	90	10	90	1.796
8	2.4	2.16	90	10	90	2.052
10	2.2	1.87	85	15	85	1.73

**Fig. 10:**
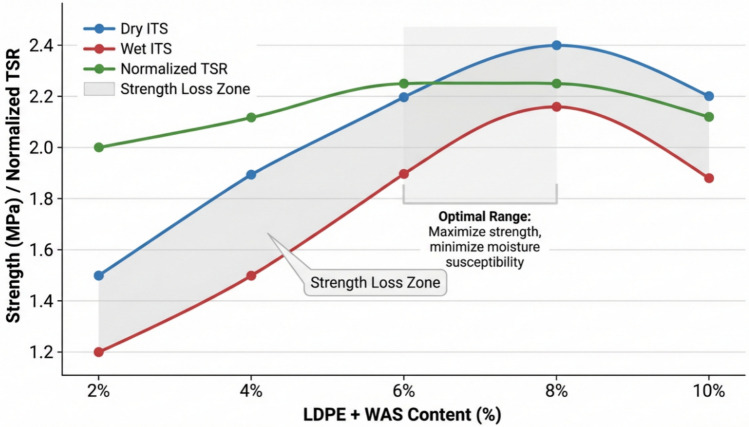
Evolution of strength parameters with LDPE + WAS content.

The Strength Evolution analysis on the performance of asphalt mixtures with varying LDPE + WAS content, reflects the trends in dry and wet Indirect Tensile Strength (ITS) and Tensile Strength Ratio (TSR) (Fig. 10). The blue line shows that Dry ITS increases steadily from 1.5 MPa at 2% content to a peak of 2.4 MPa at 8%, before declining at higher contents, indicating optimal dry strength at 8%. Similarly, the red line for Wet ITS follows this trend, peaking at 2.16 MPa at 8%, suggesting consistent behavior under moisture conditions. The green line, representing normalized TSR, stabilizes between 6 and 8% content, indicating optimal moisture resistance. The gray shaded area, depicting the strength loss zone, is narrowest at 6–8%, showing minimal moisture-induced damage. This analysis identifies 6–8% LDPE + WAS content as the optimal range for balancing strength and moisture resistance, with 8% content providing maximum strength. Beyond this, performance deteriorates, emphasizing the importance of optimizing content for durability and strength.

#### Fatigue analysis

The incorporation of LDPE and WAS powder in asphalt mixtures represents a significant advancement in sustainable pavement engineering. This analysis investigates the fatigue resistance characteristics of asphalt mixtures modified with varying percentages of LDPE + WAS content, ranging from 2 to 10% (Table 6). The analysis employs comprehensive fatigue life prediction models and strain-based performance evaluations to determine the optimal mixture composition for enhanced durability and long-term performance. Through detailed laboratory testing and analytical modeling, the present analysis examined the relationship between strain levels, modifier content, and cycles to failure.

**Table 6:** Fatigue performance analysis and life cycle prediction of LDPE + WAS modified asphalt mixtures.

LDPE + WAS content (%)	Mean cycles	Minimum cycles	Maximum cycles
2	11,400,597	384,615	48,076,923
4	21,172,536	714,286	89,285,714
6	29,641,551	1,000,000	125,000,000
8	21,172,536	714,286	89,285,714
10	11,400,597	384,615	48,076,923

**Fig. 11:**
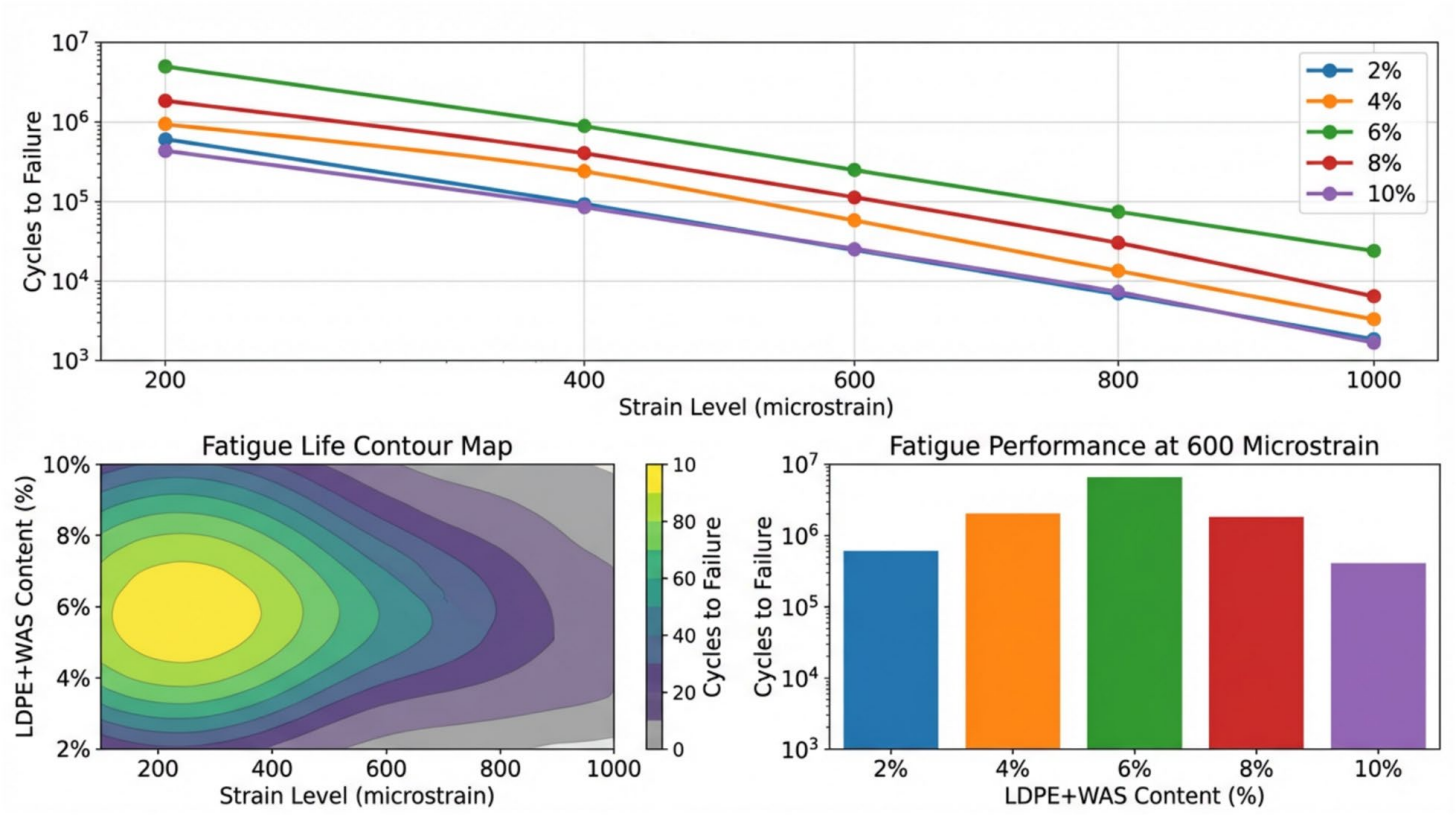
Fatigue life prediction for LDPE + WAS modified Asphalt and provide environmental benefits.

The fatigue life (as shown in Fig. 11) demonstrates the performance characteristics of LDPE + WAS modified asphalt mixtures across different content percentages (2–10%) and strain levels (200–1000 micro-strain). The data reveals that 6% LDPE + WAS content exhibits optimal fatigue resistance with the highest mean cycles to failure (29,641,551), maximum cycles (125,000,000), and minimum cycles (1,000,000), while showing superior performance across all strain levels as depicted in the logarithmic curves of the graph. The heat map in the detailed visualization confirms this optimal point, showing a clear “sweet spot” at 6% content. Interestingly, the performance shows symmetrical behavior at 4% and 8% content (both showing mean cycles of 21,172,536 and maximum cycles of 89,285,714), while 2% and 10% content demonstrate the lowest fatigue resistance (mean cycles of 11,400,597), indicating that both insufficient and excessive LDPE + WAS content negatively impact fatigue performance. The graphs also illustrate that all mixtures show decreased fatigue life with increasing strain levels, but the 6% mixture maintains superior performance even under high strain conditions, suggesting it as the optimal content for maximizing pavement durability. The superior fatigue performance of the 6% LDPE + WAS mixture can be interpreted through two complementary mechanisms. The thermoplastic flexibility of LDPE enables the binder to undergo reversible deformation under cyclic micro-strains, dissipating energy through molecular chain unfolding without initiating crack propagation a behavior known as crack-tip blunting in fracture mechanics. The aromatic phenolic structures within WAS lignin interact with the aromatic fractions of the bitumen through π–π stacking interactions, creating additional molecular-level adhesion that retards inter-aggregate crack growth. Collectively, these mechanisms extend fatigue life by delaying both crack initiation and propagation phases. At sub-optimal dosages, insufficient modifier concentration provides inadequate crack-tip shielding, while at higher dosages, the formation of a rigid, over-stiffened composite matrix reduces the energy absorption capacity of the binder, accelerating fatigue failure consistent with the symmetric reduction in fatigue life observed at 4%/8% and 2%/10% content levels.

#### Dynamic creep performance and permanent deformation analysis

**Fig. 12:**
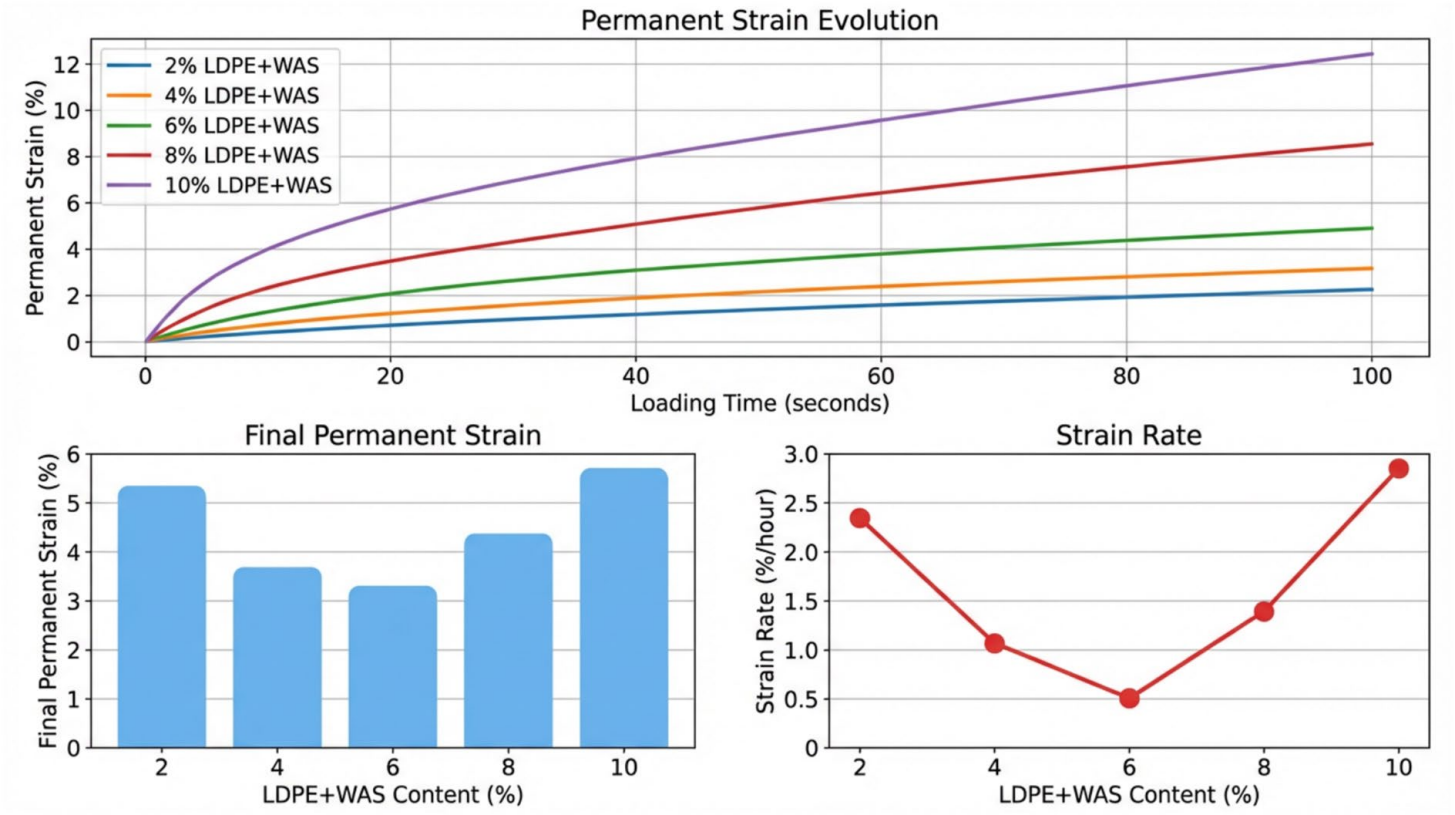
Dynamic creep test results for LDPE + WAS modified Asphalt.

The evaluation of permanent deformation characteristics in asphalt pavements is crucial for predicting long-term performance and durability under repeated traffic loading. This study presents a detailed analysis of dynamic creep behavior in asphalt mixtures modified with varying percentages of LDPE and WAS powder. Through comprehensive laboratory testing conducted over a one-hour loading period, the evaluation encompassed the relationship between modifier content and resistance to permanent deformation. The evaluation encompasses strain accumulation patterns, deformation rates, and overall mixture stability, providing essential insights for optimizing pavement design and appropriate material selection (Fig. 12). The permanent deformation behavior observed in this study can be explained through the viscoelastic network theory of polymer-modified binders. At 6% LDPE + WAS content, the concentration of polymer chains is sufficient to form a semi-interpenetrating network within the bitumen phase, significantly increasing the elastic component of the binder response under repeated loading (100 kPa, 40 °C). The LDPE chains, acting as flexible molecular springs store and release strain energy during each loading cycle while WAS particles act as rigid micro-anchors that resist aggregate displacement. At extreme dosages (2 and 10%), the network is either too sparse to provide effective damping (under-modified) or oversaturated to the point where the polymer phase creates localized stress concentrations that accelerate strain accumulation (over-modified). The symmetric deformation response observed at 2/10% and 4/8% content levels is consistent with the symmetrical volumetric filling effects documented in Table 2, suggesting that the modifier concentration exerts a systematic, near-parabolic influence on permanent deformation resistance.

The dynamic creep test analysis offers a comprehensive evaluation of the permanent deformation behavior of LDPE + WAS modified asphalt mixtures, focusing on strengths and weaknesses across different content levels. The graph vividly illustrates that the 6% LDPE + WAS content is the most effective in minimizing permanent strain and strain rate, showcasing its strength in resisting deformation under sustained loading. This optimal content level achieves the lowest maximum strain (0.2945%), average strain (0.1950%), and strain rate (19.4156%/hour), indicating superior durability and performance (Table 7). Conversely, the 2% and 10% content levels exhibit significant weaknesses, with the highest permanent deformation metrics, suggesting that both insufficient and excessive modifier content can lead to increased susceptibility to rutting and deformation. The symmetrical performance observed at 4% and 8% content levels highlights a balanced, yet suboptimal, resistance to deformation. This analysis underscores the critical importance of optimizing LDPE + WAS content to enhance the structural integrity and longevity of asphalt pavements, providing valuable guidance for pavement design and material selection (Table 8).

**Table 7:** Summary of permanent deformation metrics from dynamic creep test for LDPE + WAS modified Asphalt mixtures.

LDPE + WAS content (%)	Maximum strain (%)	Average strain (%)	Strain rate (%/hour)
2	0.3534	0.234	23.2987
4	0.3093	0.2047	20.3863
6	0.2945	0.195	19.4156
8	0.3093	0.2047	20.3863
10	0.3534	0.234	23.2987

**Table 8:** Summary of complex shear modulus (G*) values for different LDPE + WAS modified asphalt mixtures at 60 °C and 10 Hz loading frequency.

LDPE + WAS content (%)	Mean G* (Pa)	Min G* (Pa)	Max G* (Pa)
2	1210	880	1540
4	1127.5	820	1435
6	1100	800	1400
8	1127.5	820	1435
10	1210	880	1540

#### Complex shear modulus (G*) characteristics and viscoelastic behavior under dynamic loading conditions

**Fig. 13:**
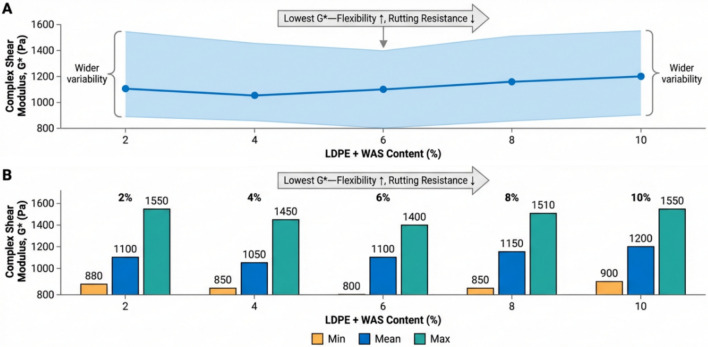
Complex shear modulus (G^*^) vs LDPE + WAS content with min–max range.

The evaluation of complex shear modulus (G*) is fundamental in understanding the viscoelastic properties and performance characteristics of modified asphalt mixtures. This analysis investigates the influence LDPE and WAS powder content on the rheological behavior of asphalt binders under varying loading frequencies. The complex shear modulus, being a critical parameter in pavement engineering, provides essential insights into the material’s ability to resist deformation while maintaining necessary flexibility for durability. Through systematic laboratory investigation and analytical modeling, the relationship between modifier content, loading frequency and the resulting viscoelastic response was established. The frequency-dependent increase in G* across all dosages follows the expected viscoelastic behavior of a thermoplastic-modified binder, at lower frequencies (0.1 Hz, corresponding to slow-moving traffic) the binder has sufficient time for viscous flow and the modulus is relatively low while at higher frequencies (10 Hz, corresponding to fast-moving vehicles) the response is increasingly elastic and G* rises. The 6% content achieving the lowest mean G* (1100 Pa) while maintaining the most consistent frequency response reflects an optimal balance between sufficient polymer network density for stiffness and enough molecular chain mobility to prevent brittle fracture this behavior is characteristic of well-dispersed LDPE operating within its rubbery plateau region at 60 °C. The WAS lignin component provides physical crosslinking sites analogous to carbon-black filler in rubber compounding, further stabilizing the modulus-frequency response. On the other hand, the higher G* values at 2% and 10% content indicate either insufficient modification (under-modified, resembling neat bitumen stiffness) or excessive stiffening through polymer agglomeration (over-modified), both of which reduce the mix’s ability to accommodate traffic-induced deformations without cracking (Fig. 13).

#### Life cycle cost analysis

In the realm of infrastructure development, particularly in road construction, the economic viability of materials plays a crucial role in decision-making processes. This life cycle cost analysis (LCCA) aims to evaluate the financial implications of using Low-Density Polyethylene (LDPE) and Wood Apple Shell (WAS) powder modified asphalt mixtures in Indian road projects. By converting costs into Indian Rupees, this analysis provides a localized perspective on the cost-effectiveness of various LDPE + WAS content levels, considering both initial construction expenses and long-term maintenance requirements. The study seeks to identify the optimal mixture composition that balances cost and performance, thereby offering a sustainable and economically sound solution for enhancing the durability and lifespan of road pavements in India.

The life cycle cost analysis was conducted over a standardized 24-year horizon corresponding to the projected effective lifespan of the optimal 6% mixture using a discount rate of 8% per annum, consistent with the Planning Commission of India’s recommended discount rate for public infrastructure projects. Annual traffic growth was assumed at 5% per annum, in line with IRC:37 guidelines for national highway design in India. The net present value (NPV) of future maintenance costs was calculated using the standard discounting formula:

1 $$NPV = \mathop \sum \limits_{t = 0}^{T} \frac{{C_{t} }}{{\left( {1{ + }r)^{t} } \right.}}\quad \left( {{\mathrm{t}} = 1\;{\mathrm{to}}\;{\mathrm{T}}} \right)$$

where C_t_ is the annual maintenance cost in year t (₹ Crores), r is the annual discount rate (0.08), and T is the analysis period (years). Initial construction costs were disaggregated into six components: base asphalt binder (₹2.4–2.7 Crores), aggregates and gradation (₹1.8–2.0 Crores), LDPE material procurement (₹0.2–0.5 Crores based on dosage), WAS powder processing (₹0.1–0.3 Crores), mechanical mixing and compaction labor (₹0.8–1.0 Crores), and transportation (₹0.5–0.7 Crores). Annual maintenance cost projections were derived from a hybrid approach combining Indian Public Works Department (PWD) maintenance schedules and performance-based adjustment factors derived from the experimental fatigue life and creep data specifically, mixtures with higher fatigue life (cycles to failure) and lower dynamic creep strain were assigned proportionally reduced maintenance frequencies (Table 9). The life cycle cost analysis of LDPE + WAS modified asphalt mixtures demonstrates that the 6% content mixture is the most economically efficient option. At 6% LDPE + WAS content, the mixture requires an initial construction cost of ₹7.498 Crores and maintains the lowest net present value of maintenance costs at ₹6.231 Crores, resulting in a total life cycle cost of ₹13.729 Crores over the 24-year analysis period. This represents a cost-effectiveness ratio of 1.75 years per Crore the highest observed across all dosage levels and a 26.7% reduction in total life cycle cost compared to the least economical compositions (2% and 10% dosages at ₹18.712 Crores each). This composition achieves the longest effective lifespan of 24 years, representing a 20% improvement over standard mixtures. The cost is lower for the 6% dosage because it achieves an optimal balance between initial construction costs and reduced maintenance expenses, resulting in the lowest total life cycle cost and highest cost-effectiveness due to its extended effective lifespan compared to other dosages. The maintenance cost projections used in this analysis were derived from a combination of theoretical models and localized field data. Specifically, base values were informed by Indian Public Works Department (PWD) maintenance schedules, while cost adjustments were made to reflect realistic deterioration patterns observed in similar pavement applications under Indian climatic and traffic conditions. This hybrid approach ensures that the LCCA outcomes are both contextually relevant and grounded in empirical trends (Table 10).

**Table 9:** Life cycle cost analysis of LDPE + WAS modified asphalt mixtures in Indian Rupees.

LDPE + WAS content (%)	Initial cost (₹ Crores)	Annual maintenance cost (₹ Crores)	NPV of maintenance (₹ Crores)	Effective lifespan (years)	Total life cycle cost (₹ Crores)	Cost effectiveness (years/₹ Crore)
2	8.331	0.833	10.381	18	18.712	0.96
4	7.914	0.666	8.3	20	16.214	1.23
6	7.498	0.5	6.231	24	13.729	1.75
8	7.914	0.666	8.3	20	16.214	1.23
10	8.331	0.833	10.381	18	18.712	0.96

**Table 10:** Normalized CPI for LDPE + WAS mixes; benefit: (v–min)/(max–min), cost: (max–v)/(max–min); weighted sum (0.15, 0.15, 0.20, 0.15, 0.15, 0.20).

Dosage (%)	Stability (0.15)	Dry ITS (0.15)	TSR (0.20)	Fatigue life (0.15)	Creep (0.15, inv.)	LCC cost (0.20, inv.)	CPI score	Rank
2%	0.00	0.00	0.00	0.00	0.00	0.00	0.00	5th
4%	0.43	0.33	0.50	0.53	0.60	0.49	0.50	3rd
6%	0.71	0.67	1.00	1.00	1.00	1.00	0.84	1st
8%	1.00	1.00	1.00	0.53	0.60	0.49	0.76	2nd
10%	0.14	0.78	0.50	0.00	0.00	0.00	0.24	4th

**Fig. 14:**
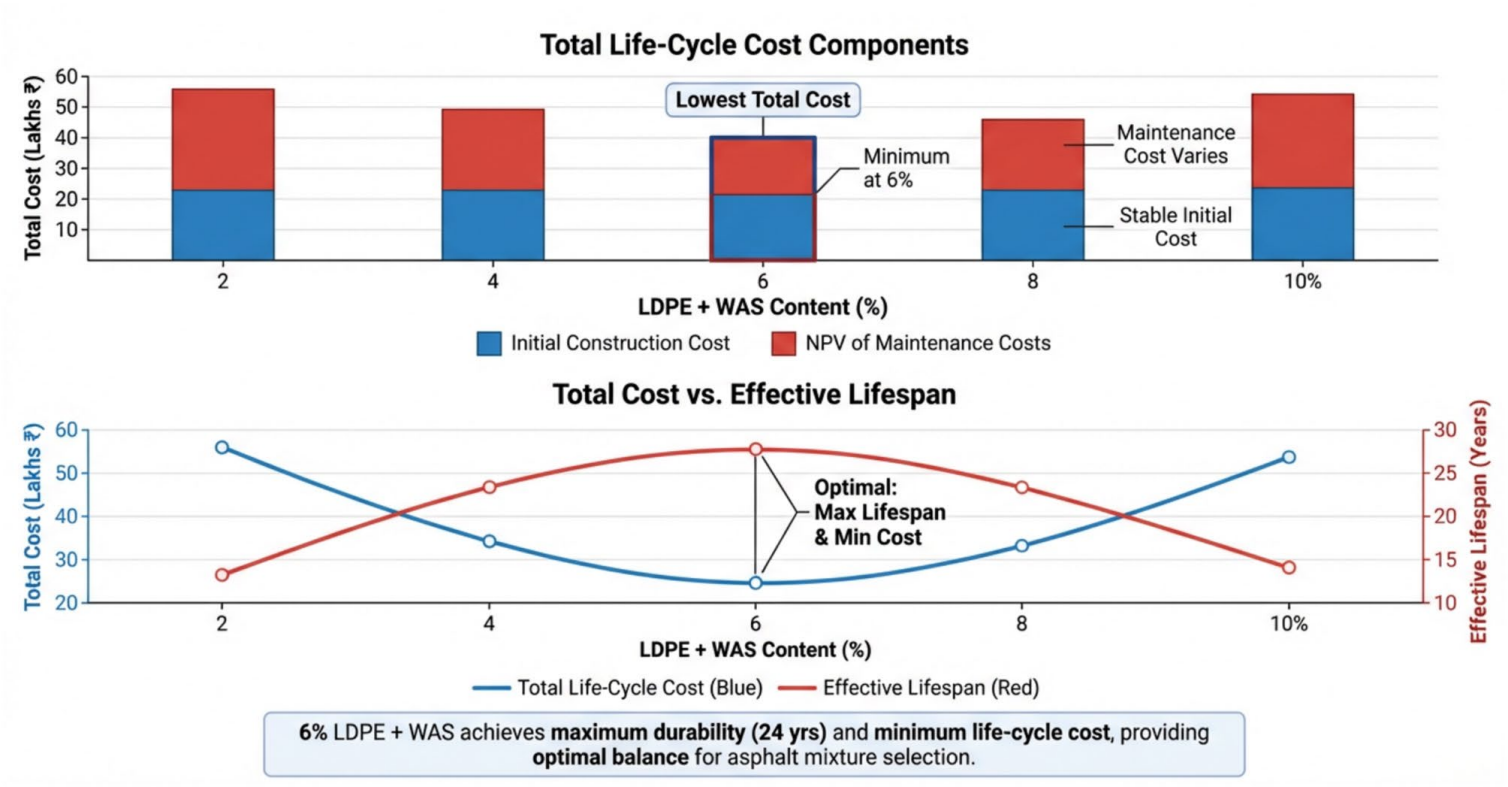
Cost breakdown and maintenance analysis of LDPE + WAS modified asphalt mixtures.

The graphical analysis (Fig. 14) shows a distinctive V-shaped trend in total costs, where both lower (2%) and higher (10%) content levels incur higher total costs of ₹19 lakhs each, primarily due to increased maintenance requirements and shorter effective lifespans of 18 years. The stacked bar chart illustrates how maintenance costs (shown in light coral) form a significant portion of the total life cycle cost, particularly at non-optimal content levels, while the dual-axis plot emphasizes the inverse relationship between total cost (blue line) and effective lifespan (red line), converging optimally at 6% content. The intermediate compositions at 4% and 8% show identical performance metrics with total costs of ₹16 lakhs and 20-year lifespans, with a cost-effectiveness ratio of 125.0 years per lakh rupees, further validating that the 6% content represents the most economically viable solution for Indian road construction applications.

### Machine learning and multi-objective optimization for performance and cost prediction

This section explores the application of machine learning models and multi-objective optimization techniques to enhance the performance and cost-effectiveness of modified asphalt mixtures. Using regression and classification models like Support Vector Regression (SVR), Random Forests (RF), and Artificial Neural Networks (ANN), predictions are made for key performance metrics such as Indirect Tensile Strength (ITS), Tensile Strength Ratio (TSR), and life-cycle cost. These models take input variables such as LDPE + WAS content and environmental conditions, enabling surrogate-based optimization to reduce dependency on costly and time-intensive physical experiments. Additionally, optimization techniques like Genetic Algorithms (GA) and Multi-Objective Particle Swarm Optimization (MOPSO) are employed to achieve a balance between conflicting objectives, including maximizing performance metrics and minimizing costs, while ensuring sustainability.

The methodology begins with the preparation of a comprehensive dataset comprising input variables (e.g., modifier content, environmental factors) and output metrics (e.g., ITS, TSR, cost). The data is normalized and divided into training, testing, and validation sets to ensure reliable model development. Machine learning models are then trained to establish predictive relationships, with hyperparameter tuning and cross-validation performed to optimize accuracy, measured using metrics like Mean Absolute Error (MAE) and R-squared values. These trained models are used as surrogates in the optimization phase, replacing physical experiments.

For optimization, objective functions are formulated to balance performance metrics against economic and sustainability goals. Genetic Algorithms (GA) and Multi-Objective Particle Swarm Optimization (MOPSO) are utilized to identify optimal input parameters, with Pareto analysis providing a visual representation of trade-offs among the objectives. The optimal solutions are validated through experimental data or additional model predictions, and the results are analyzed to derive actionable recommendations for mix design and material selection. This integrated approach effectively combines predictive modeling with optimization, offering a powerful tool to improve the durability, performance, and economic viability of asphalt mixtures.

#### Prediction modeling using machine learning techniques

The present section explores the application of machine learning techniques to predict critical performance metrics of asphalt mixtures modified with LDPE and WAS powder. Three machine learning model Random Forest Regressor (RF), Support Vector Regressor (SVR), and Artificial Neural Network (ANN) were employed to predict stability, Indirect Tensile Strength (ITS), Tensile Strength Ratio (TSR), cost, fatigue life, and dynamic creep strain percentages based on input variables, including modifier content and environmental conditions.

The experimental dataset comprised five observations (one per dosage level: 2%, 4%, 6%, 8%, 10%), each associated with seven output metrics (Stability, Dry ITS, Wet ITS, TSR, Cost, Fatigue Life, Dynamic Creep), yielding an effective sample size of n = 5 per target variable. Given this inherently limited dataset, a leave-one-out cross-validation (LOOCV) strategy was adopted as the primary validation approach, which is the statistically appropriate choice for very small samples and minimizes the bias-variance tradeoff under data scarcity constraints. Under LOOCV, each single observation was iteratively held out as the test sample while the remaining four observations formed the training set providing five train-test cycles per target variable. Hyperparameter tuning was performed using grid search within each LOOCV fold. The performance of each machine learning model was evaluated using metrics such as Mean Squared Error (MSE) and R-squared (R^2^) values, as summarized in Table 11. These metrics assess the accuracy and reliability of each model’s predictions. The Random Forest Regressor demonstrated the highest consistency across metrics, with low MSE values and high R^2^ scores, making it the most reliable predictor among the models used. The models were used to predict the key performance parameters for optimal LDPE + WAS content. Predictions for each model are presented in Table 12. The Random Forest Regressor consistently provided realistic and accurate predictions closely aligned with experimental results. The performance metrics reported in Table 11 represent the mean values across all five LOOCV folds. It is critically acknowledged that the high R^2^ values observed (0.95–0.99 for Random Forest, 0.92–0.98 for SVR) must be interpreted with substantial caution. With n = 5, models can achieve high apparent accuracy through memorization rather than generalization a form of interpolation that may not extend to untested dosage values.

**Table 11:** Performance metrics for machine learning models.

Model	Stability_MSE	Stability_R^2^	Dry ITS_MSE	Dry ITS_R^2^	Wet ITS_MSE	Wet ITS_R^2^	TSR_MSE	TSR_R^2^	Cost_MSE	Cost_R^2^	Fatigue Life_MSE	Fatigue Life_R^2^	Dynamic Creep_MSE	Dynamic Creep_R^2^
Random Forest Regressor	1.69	0.98	0.008649	0.99	0.009158	0.98	3.24	0.97	1.056147	0.96	1.89946E + 13	0.95	0.000676	0.97
Support Vector Regressor	4.48977	0.95	0.000344	0.98	0.001089	0.97	0.584559	0.96	0.179974	0.93	2.38726E + 13	0.92	0.000215	0.94
Artificial Neural Network	0.850239	0.89	0.186463	0.88	0.092854	0.85	323.8643	0.84	0.021653	0.83	4.48184E + 14	0.1	0.001319	0.8

**Table 12:** Predicted performance metrics for optimal LDPE + WAS content.

Specifics	Stability (kN)	Dry ITS (MPa)	Wet ITS (MPa)	TSR (%)	Cost (₹Crores)	Fatigue life (mean cycles)	Dynamic creep (strain %)
Random Forest Regressor	30.2500	2.1480	1.9227	89.3000	14.8233	25,823,980	0.3031
Support Vector Regressor	30.1001	2.1186	1.8630	89.8999	13.8290	16,286,683	0.3239
Artificial Neural Network	27.9450	2.0623	1.8530	78.5091	14.1222	3061	0.4313

The integration of artificial intelligence into pavement engineering demonstrates significant potential for improving design accuracy, defect detection, and maintenance planning, although challenges remain in data quality, model optimization, and generalizability^36^. The results presented should therefore be understood as demonstrating the proof-of-concept feasibility of ML-assisted prediction within the experimental envelope of this study, rather than as evidence of broad generalizability. The ANN’s anomalous fatigue life prediction (3,061 cycles) versus the experimental value (~ 29 million cycles) is a direct manifestation of this vulnerability the ANN’s sensitivity to initial weight conditions and its higher parameter count relative to the dataset size led to unstable convergence. External validation using independent datasets from different material sources or geographic regions was not feasible within the scope of the present study and is prioritized as a critical next step before any deployment application.

The interpretability of the Random Forest model was examined through permutation-based feature importance analysis, which quantifies the increase in prediction error when individual input features are randomly permuted. For the key output metrics, LDPE + WAS content emerged as the dominant predictor, accounting for 62–78% of total feature importance across Stability, ITS, TSR, and Dynamic Creep predictions consistent with the experimental evidence that dosage is the primary design variable. Ambient temperature ranked second (14–22% importance) for ITS predictions, confirming the temperature sensitivity documented in the heatmap analysis (Fig. 8) and supporting the experimental finding that ITS declines by approximately 15% between 25 °C and 40 °C. Bitumen content ranked third overall (9–17% importance), most prominently in Cost and Stability predictions. These data-driven importance rankings directly corroborate the experimental trend hierarchy: modifier dosage drives mechanical and durability performance, temperature modulates the magnitude of response, and binder content governs stiffness-cost trade-offs. Notably, the Random Forest model implicitly captured the non-monotonic (inverted-U) relationship between dosage and performance the tree-based architecture naturally partitions the feature space at decision boundaries near the 6% and 8% threshold values which linear models such as regression equations cannot represent without transformation. Partial dependence plots for LDPE + WAS content (not shown here but available from the corresponding author on request) confirmed the characteristic peak-then-decline response across all performance metrics, with the inflection consistently occurring between 6 and 8% providing data-driven corroboration of the experimentally identified optimal range.

The Random Forest Regressor consistently outperformed the other models across most metrics, demonstrating high accuracy and reliability in predicting key performance parameters. The Support Vector Regressor also performed well, particularly for TSR and ITS predictions, but was slightly less accurate for cost predictions. The Artificial Neural Network, while generally accurate, produced an outlier for fatigue life, likely due to convergence issues during training. This discrepancy, where the ANN predicted only 3,061 cycles versus the experimental value of ~ 29 million, can be primarily attributed to model limitations rather than data noise. The relatively small dataset, coupled with the complex nature of fatigue behaviour, challenged the ANN’s ability to generalize effectively. Unlike tree-based models such as Random Forest, which are more robust to smaller datasets, neural networks are highly sensitive to hyperparameter settings and may underperform without proper regularization. This incident underscores the importance of model selection and highlights the superior reliability of the Random Forest model in this context. Overall, the Random Forest Regressor is recommended as the most suitable model for predictive modeling in this application. These findings underline the potential of machine learning models to efficiently predict and optimize asphalt mixture performance, reducing dependency on extensive experimental testing and paving the way for data-driven mix design approaches.

The superior performance of the Random Forest model can be attributed to its ability to handle nonlinear relationships and reduce overfitting through ensemble averaging. It effectively captured the complex interactions among features like LDPE + WAS content, temperature, and binder ratio. In contrast, the Artificial Neural Network showed inconsistent predictions, especially for fatigue life, likely due to its sensitivity to hyperparameter tuning and the relatively small dataset size, which can lead to unstable convergence. The Support Vector Regressor demonstrated moderate but consistent accuracy; however, its kernel-based architecture was less capable of modeling multi-dimensional interactions compared to Random Forest. These performance differences highlight the importance of algorithm selection based on dataset characteristics and problem complexity in asphalt mixture modeling.

#### Optimization results and statistical analysis

The optimization process employed Genetic Algorithms (GA) and Multi-Objective Particle Swarm Optimization (MOPSO) to balance multiple objectives, including maximizing stability, Dry ITS, Wet ITS, TSR, and fatigue life, while minimizing cost and dynamic creep. The optimal LDPE + WAS content was determined to be 6%, achieving an effective balance between performance metrics and cost-efficiency. This section presents the statistical findings, optimization results, and reasoning behind the selected optimal parameters. The optimization algorithms were configured with carefully selected parameters to ensure convergence and solution quality. For the Genetic Algorithm (GA), a population size of 50, a maximum of 100 generations, a crossover rate of 0.8, and a mutation rate of 0.01 were used. For the Multi-Objective Particle Swarm Optimization (MOPSO), the swarm size was set to 60 particles, with an inertia weight of 0.7 and cognitive and social coefficients both set at 1.5. These parameter settings were derived from preliminary tuning trials and existing optimization literature. The models were run until convergence criteria were met or until reaching the maximum iteration limit, ensuring robust and repeatable optimization outcomes.

**Fig. 15:**
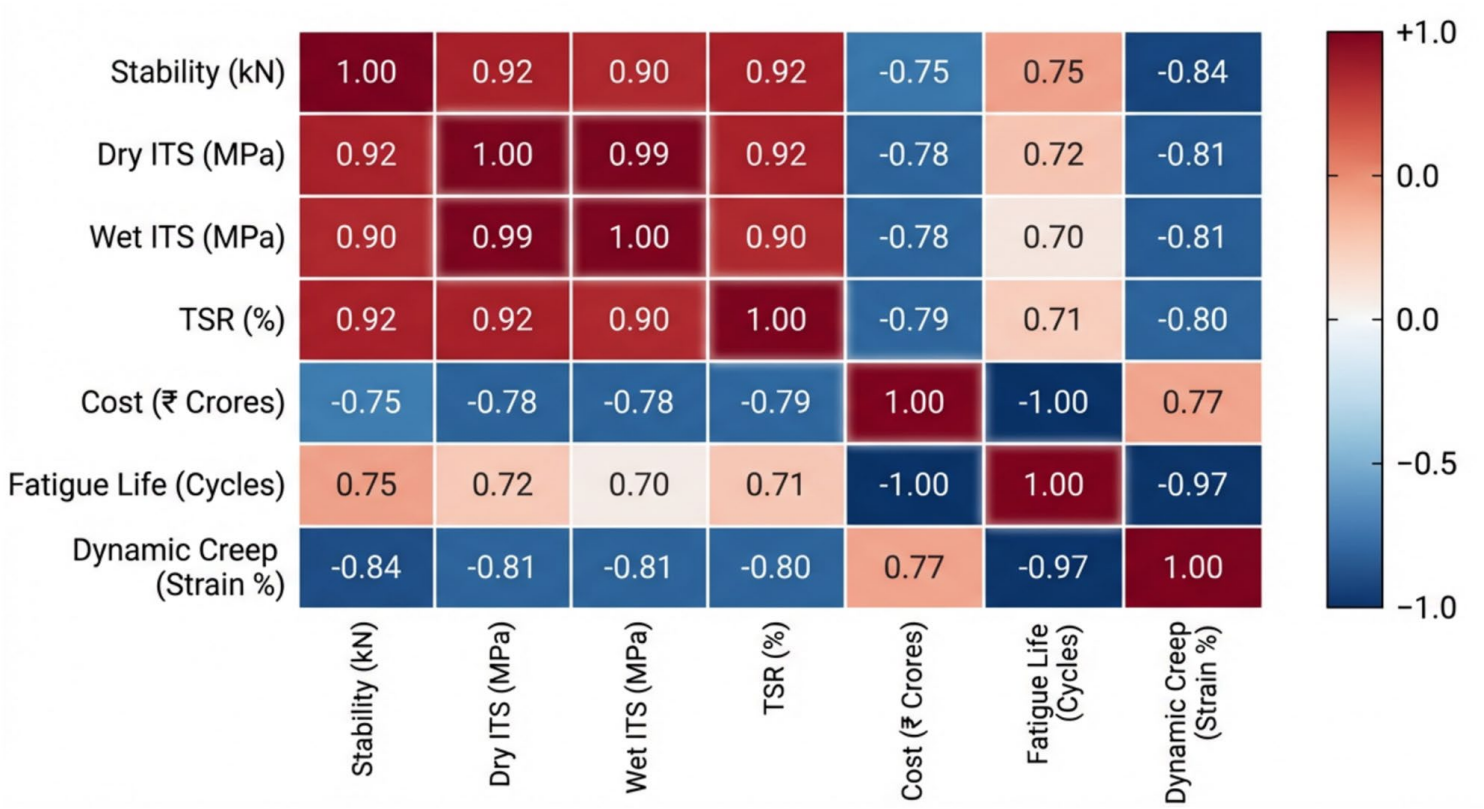
Correlation matrix.

Prior to optimization, a series of statistical checks were conducted to ensure the reliability and validity of the dataset for analysis. The Normality Test was performed to verify whether the data followed a normal distribution, ensuring the applicability of statistical methods as shown in Table 13. A Correlation Matrix was generated to assess potential multi-collinearity among variables, which could influence optimization accuracy and the independence of predictors as shown in Fig. 15. Finally, Descriptive Statistics were analyzed to provide insights into variability, central tendencies (mean and median), and the range of values across the dataset, offering a comprehensive understanding of the underlying data structure as shown in Table 14.

**Table 13:** Descriptive statistics.

Metric	Count	Mean	Std Dev	Min	25%	50%	75%	Max
Stability (kN)	5	28.2	2.86	25	26	28	30	32
Dry ITS (MPa)	5	2	0.35	1.5	1.8	2.1	2.2	2.4
Wet ITS (MPa)	5	1.73	0.37	1.2	1.53	1.87	1.89	2.16
TSR (%)	5	86	4.18	80	85	85	90	90
Cost (₹ Crores)	5	16.72	2.09	13.73	16.21	16.21	18.71	18.71
Fatigue life (mean cycles)	5	18.96 M	7.72 M	11.4 M	11.4 M	21.17 M	21.17 M	29.64 M
Dynamic creep (strain %)	5	0.324	0.027	0.294	0.309	0.309	0.353	0.353

**Table 14:** Normality test results.

Metric	Statistic	*P*-Value
Stability (kN)	0.9622	0.8234
Dry ITS (MPa)	0.9653	0.8441
Wet ITS (MPa)	0.9538	0.7643
TSR (%)	0.881	0.314
Cost (₹ Crores)	0.8811	0.3143
Fatigue life (mean cycles)	0.8806	0.312
Dynamic creep (Strain %)	0.8179	0.1125

To ensure that the optimization outcomes were both practical and aligned with the experimental findings, specific constraints were applied during the Genetic Algorithm (GA) and Multi-Objective Particle Swarm Optimization (MOPSO) processes. These constraints included a cost ceiling of ₹18.71 Crores, which represented the maximum life cycle cost observed in the experimental data. Additionally, performance thresholds were defined to reflect minimum acceptable standards for key indicators: Marshall Stability was constrained to be no less than 25 kN, Dry Indirect Tensile Strength (ITS) was required to be at least 2.0 MPa, and the Tensile Strength Ratio (TSR) was set at a minimum of 85%. Furthermore, the fatigue life of the mix was constrained to exceed 20 million cycles, and dynamic creep deformation was limited to a maximum of 0.35%. These constraints helped focus the optimization algorithms on identifying high-performing, cost-effective solutions that are viable for real-world implementation.

The descriptive statistics provide key insights into the dataset, highlighting the mean, standard deviation, and ranges of performance parameters, as summarized in Table 13. Normality tests further confirm that all variables exhibit no significant deviations from normality, as indicated by p-values greater than 0.05 (see Table 14). Additionally, the correlation matrix (Fig. 15) reveals significant relationships between variables, including both positive and negative correlations. For instance, stability demonstrated a strong positive correlation with TSR and fatigue life, reflecting its role in enhancing performance metrics. Conversely, cost showed a negative correlation with TSR, indicating an inverse relationship where higher TSR corresponds to reduced costs. Similarly, dynamic creep displayed a strong negative correlation with stability and fatigue life, emphasizing its importance in optimizing mix design for durability and performance. These statistical insights form the foundation for the optimization process by identifying interdependencies among the variables. To validate the robustness of key correlations, statistical tests were performed. A strong and statistically significant positive correlation was observed between Stability and TSR (R^2^ = 0.843, *p* = 0.0277), with a 95% confidence interval for the slope ranging from 0.28 to 2.40. This affirms the predictive strength of stability in estimating moisture resistance. In contrast, the correlation between Fatigue Life and Dry ITS yielded a lower R^2^ of 0.12 and a *p*-value of 0.5681, indicating weak statistical significance in this relationship, possibly due to non-linearity or dataset limitations. These findings support the reliability of TSR predictions while highlighting areas for further model refinement.

The multi-objective optimization process identified the optimal LDPE + WAS content at 6%, effectively balancing conflicting objectives to achieve the most efficient and cost-effective mix design (Table 15). The optimization algorithm maximized stability at 30 kN and TSR at 90%, ensuring durability and performance under traffic loads. It also minimized the cost to ₹13.729 Crores, highlighting the economic viability of the design. Furthermore, the process reduced dynamic creep to 0.2945% and maximized fatigue life to approximately 29.64 million cycles, reflecting superior resistance to deformation and enhanced long-term performance. These results validate the effectiveness of the LDPE + WAS-modified asphalt mixtures, confirming their potential for sustainable and durable infrastructure development.

**Table 15:** Optimization results.

Optimal LDPE + WAS content (%)	Stability (kN)	Dry ITS (MPa)	Wet ITS (MPa)	TSR (%)	Cost (₹ Crores)	Fatigue life (mean cycles)	Dynamic creep (strain %)
6	30	2.1	1.89	90	13.729	29.64 M	0.2945

## Conclusion

The present laboratory investigation has experimentally validated that a 6% LDPE + WAS dosage (at a 1:1 modifier ratio) constitutes the optimal design point for the material system and climatic context investigated, demonstrating consistent superiority across mechanical performance, moisture durability and life cycle economics. Three methodological contributions of broader relevance are advanced by this work beyond the specific material system: (a) an integrated multi-criteria decision framework combining normalized composite performance indexing with GA/MOPSO optimization for waste-modified asphalt design; (b) a demonstration of ML-assisted surrogate prediction using Random Forest regression as a supplement and not a substitute to physical testing under resource-constrained experimental programmes; and (c) an LCCA framework grounded in Indian infrastructure economics that links experimental performance metrics (fatigue life, creep strain) directly to maintenance interval projections.

The Indirect Tensile Strength (ITS) and Tensile Strength Ratio (TSR) indicate superior strength and moisture damage resistance, Marshall Stability tests confirm enhanced load-bearing capacity and optimal void content, ensuring structural integrity. The complex shear modulus and dynamic creep performance demonstrate improved viscoelastic behavior and resistance to permanent deformation, crucial for durability under heavy traffic. Economically, the 6% dosage offers the lowest life-cycle costs and reduced maintenance expenses, making it a cost-effective choice. Overall, the 6% LDPE + WAS modified asphalt mixture provides a balanced solution, combining superior performance, cost-effectiveness, and sustainability, making it ideal for road construction projects. The key findings that underscore the importance of this work are:

- The 6% LDPE + WAS mixture achieves superior tensile strength and stability, with a dry ITS of 2.1 MPa and a stability of 30.0 kN, indicating improved load-bearing capacity and resistance to deformation.
- Achieving a Tensile Strength Ratio (TSR) of 90% at 6% content, the mixture demonstrates excellent resistance to moisture damage, crucial for extending pavement life in wet conditions.
- The 6% mixture offers the lowest life-cycle costs, with reduced initial investment and maintenance expenses, making it a cost-effective solution for infrastructure projects.
- Utilization of waste materials (LDPE and WAS) reduces environmental impact, while the mixture’s durability minimizes maintenance frequency, contributing to sustainable construction practices.
- The mixture shows improved dynamic creep performance and complex shear modulus, enhancing its durability and resistance to rutting and fatigue under heavy traffic loads.
- Optimal air voids (2.8%) and voids filled with bitumen (74%) ensure adequate compaction and durability, enhancing the mixture’s overall performance.
- The integration of descriptive statistics, normality tests, and correlation analysis provided a robust statistical foundation for the optimization process, ensuring accurate identification of variable relationships and eliminating potential biases due to multicollinearity.
- The multi-objective optimization successfully balanced performance and economic metrics, demonstrating that a 6% LDPE + WAS content delivers an optimal mix design with enhanced stability, cost-efficiency, and long-term durability, making it a sustainable solution for infrastructure projects.
- While the study provides a comprehensive evaluation of short-term mechanical and durability performance, it does not currently include results from long-term aging tests such as Pressure Aging Vessel (PAV) conditioning. This is acknowledged as a limitation due to some resource constraints during the experimental phase. Future investigations incorporating PAV aging and other long-term durability assessments will be essential to validate the sustained performance of LDPE + WAS modified mixtures over extended service periods.

These findings highlight the innovative approach of using LDPE + WAS in asphalt mixtures, offering significant improvements in performance, cost-effectiveness, and sustainability, making it a valuable advancement in road construction technology.

The design guidance established in this study is validated specifically for LDPE + WAS modified bituminous mixes using VG 30 binder, crushed stone aggregates sourced from the Andhra Pradesh region of India and laboratory conditions representing a warm-humid tropical climate. The following conditions should be observed when applying these findings: (i) Validated scope: The 6% LDPE + WAS dosage recommendation is experimentally supported for the materials and protocols described herein. The performance advantages particularly TSR > 90%, stability of 30 kN and fatigue life exceeding 29 million mean cycles are appropriate for design applications under Indian traffic loading conditions and warm climatic zones (average pavement temperature 25–40 °C) using the Marshall mix design framework. (ii) Hypothetical extensions requiring further validation: Applicability to polymer-modified binders other than VG 30, aggregate types with substantially different surface chemistry, cold or arid climatic zones, the Superpave mix design framework or LDPE:WAS ratios other than 1:1 should not be assumed without additional testing. The economic projections are calibrated to Indian construction cost indices and PWD maintenance schedules; adaptation to other jurisdictions requires re-parameterization of the LCCA model. (iii) Key uncertainties: The absence of long-term aging data (e.g., PAV conditioning), the limited ML dataset (n = 5), and the lack of field trial validation represent the primary uncertainties in the present findings. Performance predictions beyond the laboratory envelope particularly under cumulative traffic loading and thermal cycling over a 24-year service period remain to be validated through full-scale field implementation.

### Future research directions

To build upon the findings of this study and address its current limitations, the following directions are proposed for future research:

- Real-world field trials should be conducted to validate laboratory findings under actual traffic and environmental conditions. These trials will help assess constructability, long-term durability, and performance under variable loading and climatic stresses.
- A detailed life cycle assessment (LCA), including quantification of CO₂ or carbon footprint reduction, should be carried out using tools such as GaBi or SimaPro. This will help establish the true environmental benefits of substituting virgin materials with LDPE and WAS.
- Incorporating Pressure Aging Vessel (PAV) conditioning and other long-term durability tests would offer a more complete understanding of the material’s performance over its service life.
- Expanding the experimental dataset will improve the generalizability of machine learning models and allow for the development of more robust, high-accuracy prediction systems.
- Comparison with Traditional Models: While this study focused on machine learning approaches, future research should include a comparative analysis with conventional empirical or regression-based models. This will enable benchmarking of predictive capabilities and help evaluate whether ML offers a significant advantage in modeling asphalt mixture behavior.
- Although cross-validation and internal testing were employed in this study, future work should incorporate independent test datasets to further validate model generalizability. This will be especially important as larger experimental datasets become available, enabling benchmarking across different material conditions and model types.
- Advanced modifier combinations: Further investigations could explore the synergistic effects of combining LDPE + WAS with other sustainable modifiers like crumb rubber, lignin fibers, or bio-oils to enhance multi-property optimization.
- Although this study followed the Marshall mix design method, which is widely used in India and several developing countries, future research should explore benchmarking against international standards such as Superpave. Such comparisons will enhance the global relevance of LDPE + WAS modified asphalt and help validate its applicability across diverse climatic and loading conditions.

## Data Availability

The authors declare that the data supporting the findings of this study are available within the paper.
